# Supplementary figures and images for: Targeting PI3K/Akt/mTOR signaling in rodent models of PMP22 gene-dosage diseases
Source: EMBO Mol Med. 2024 Feb 21;16(3):616–40. doi: 10.1038/s44321-023-00019-5 (PMC10940316; doi:10.1038/s44321-023-00019-5)

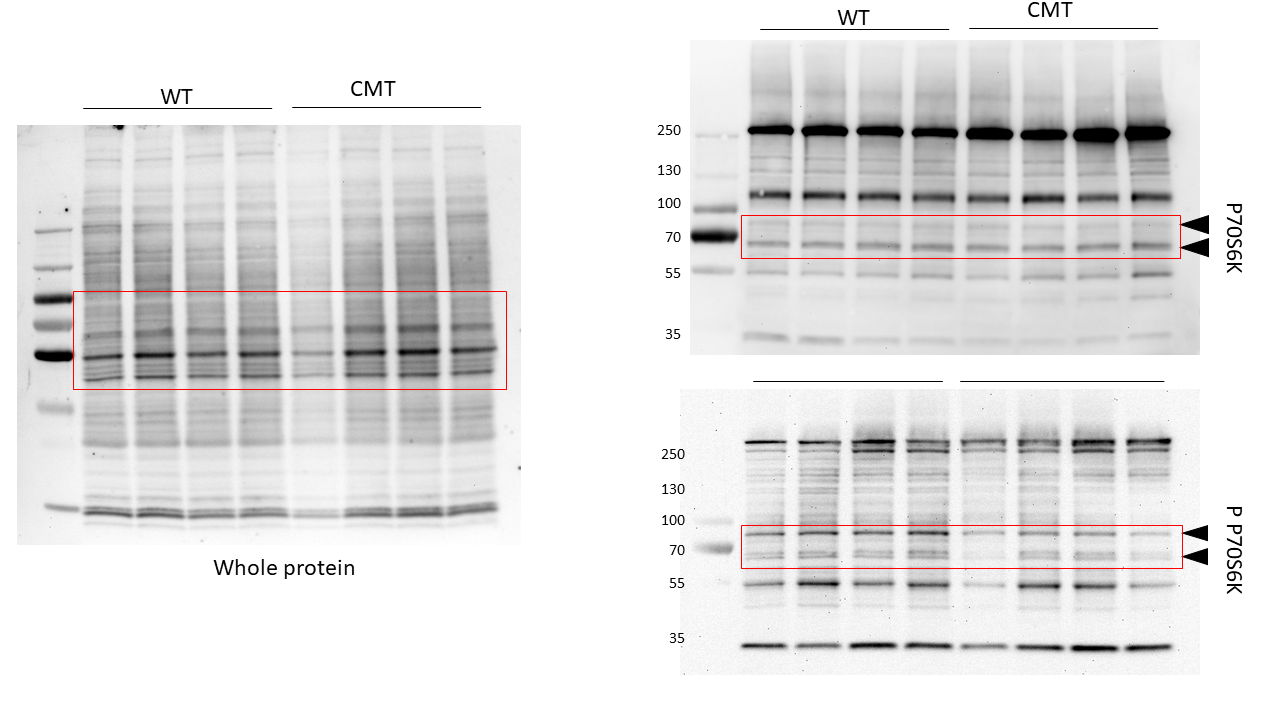

Supplement: Supplementary file 1 — Source Data Fig. 1 [file 44321_2023_19_MOESM1_ESM.zip › Figure1/1D_Blot/PhosphoBlot.tif]

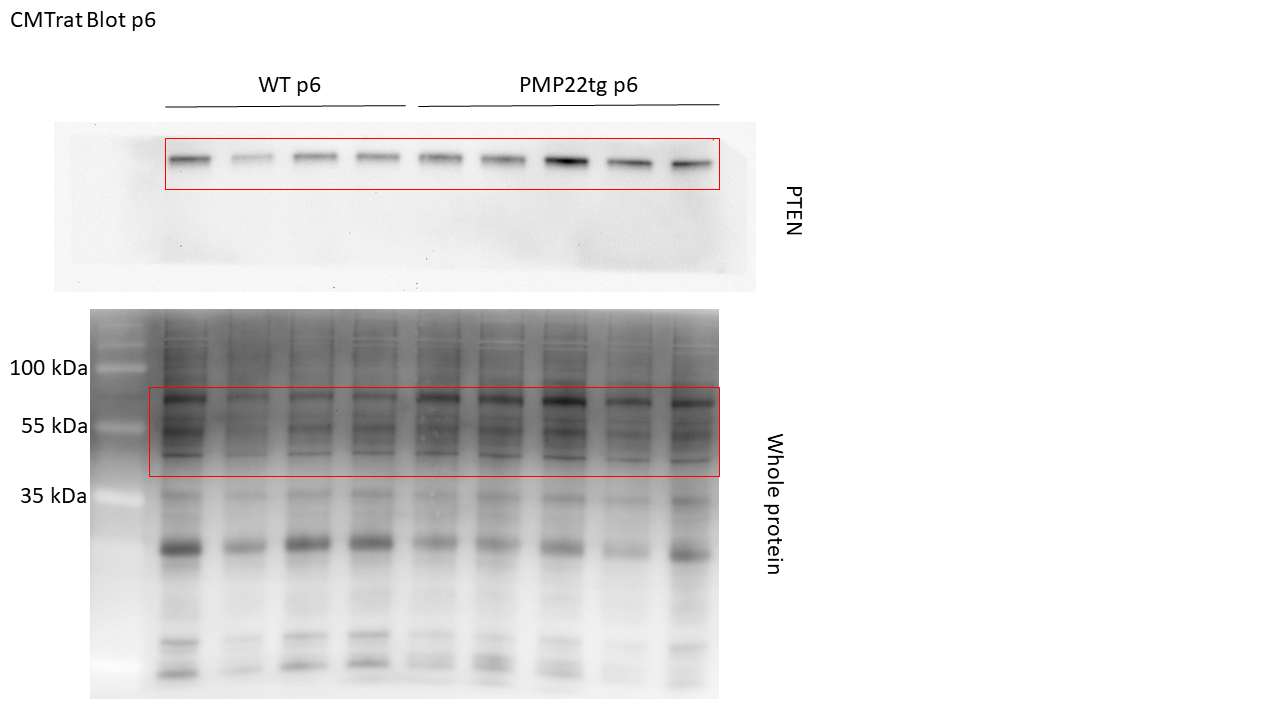

Supplement: Supplementary file 1 — Source Data Fig. 1 [file 44321_2023_19_MOESM1_ESM.zip › Figure1/1D_Blot/PTENP6CMT.tif]

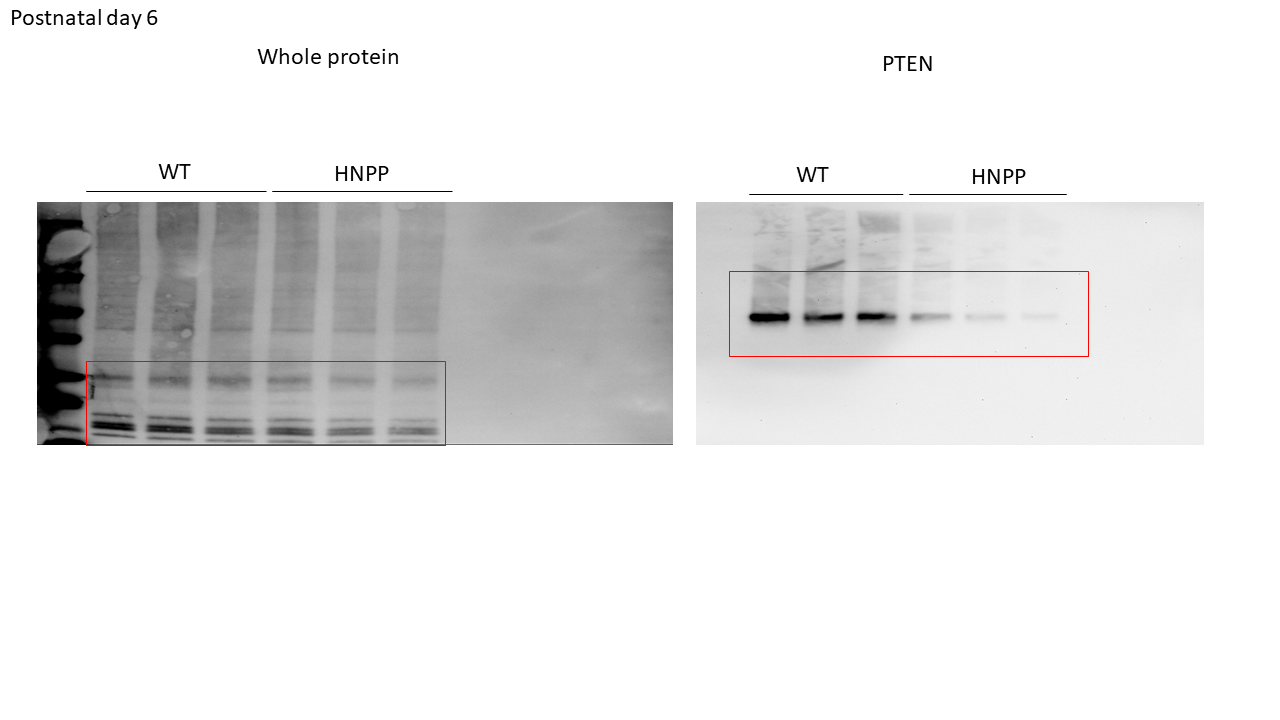

Supplement: Supplementary file 1 — Source Data Fig. 1 [file 44321_2023_19_MOESM1_ESM.zip › Figure1/1B_Blot/PTENP6HNPP.tif]

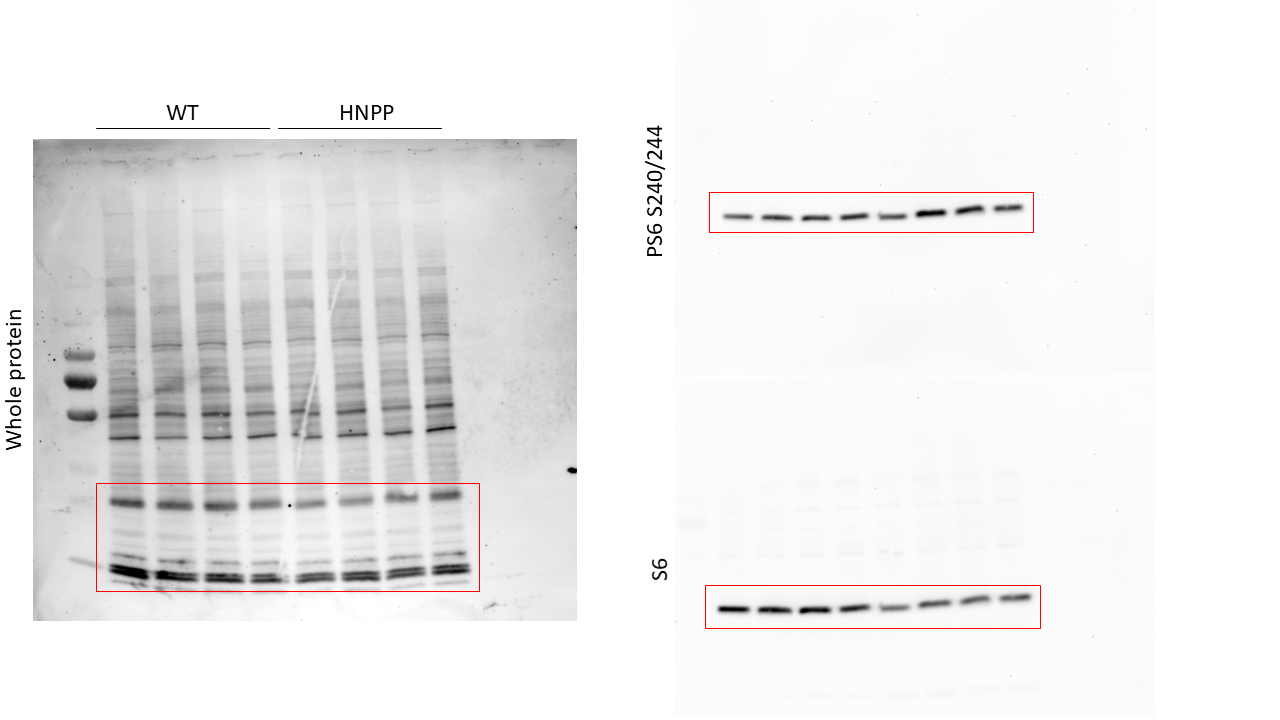

Supplement: Supplementary file 1 — Source Data Fig. 1 [file 44321_2023_19_MOESM1_ESM.zip › Figure1/1B_Blot/PS6P6HNPP.tif]

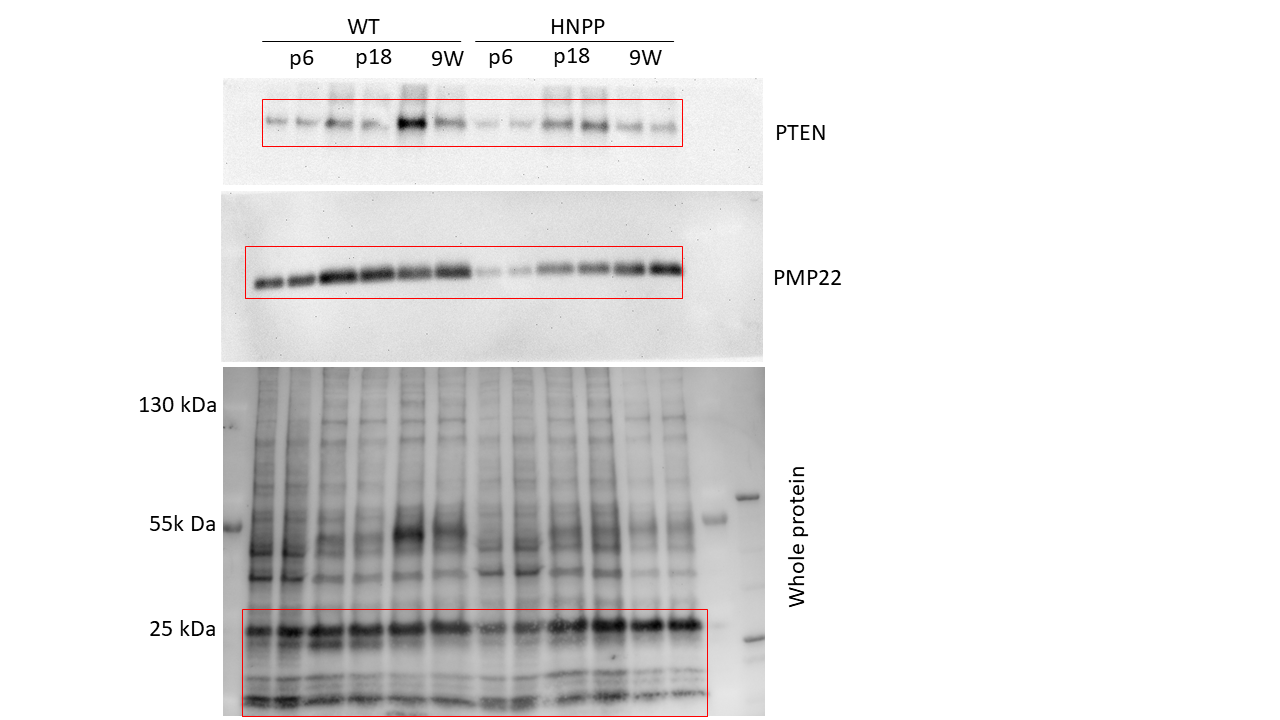

Supplement: Supplementary file 1 — Source Data Fig. 1 [file 44321_2023_19_MOESM1_ESM.zip › Figure1/1A_Blot/Blot_HNPPtimeline.tif]

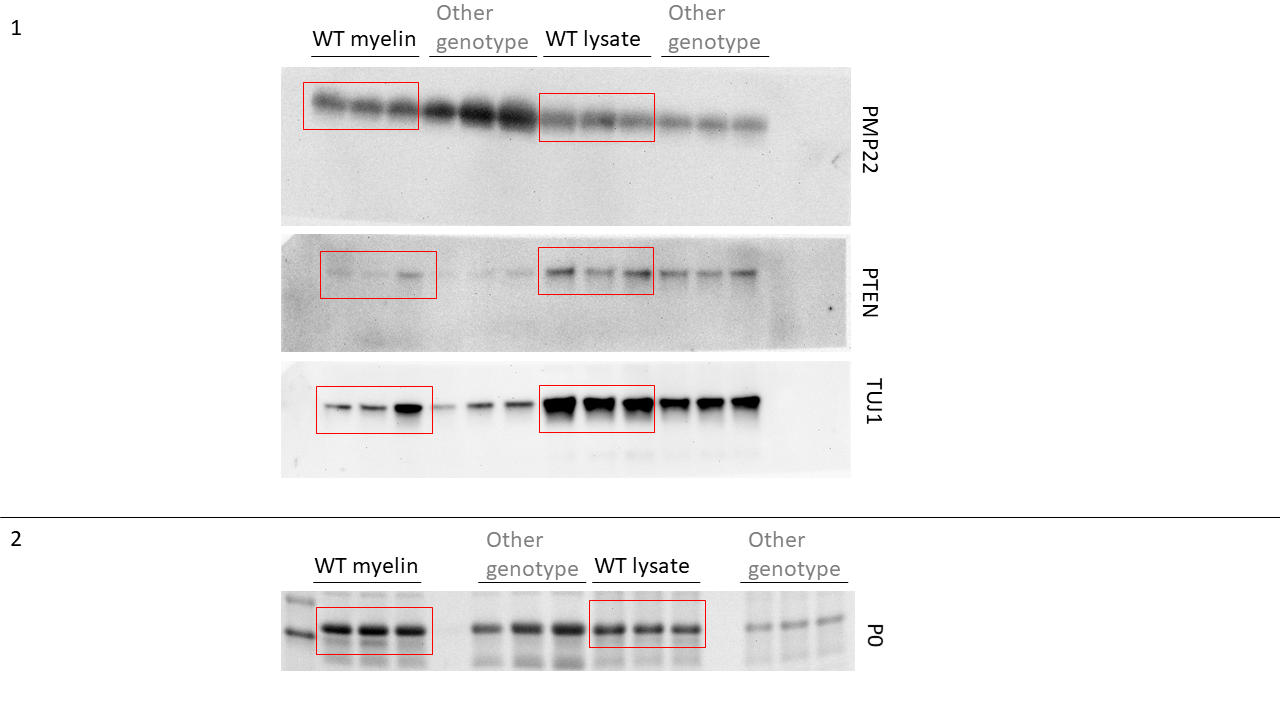

Supplement: Supplementary file 1 — Source Data Fig. 1 [file 44321_2023_19_MOESM1_ESM.zip › Figure1/1G_Blots/1G_MyelinLysateWT.tif]

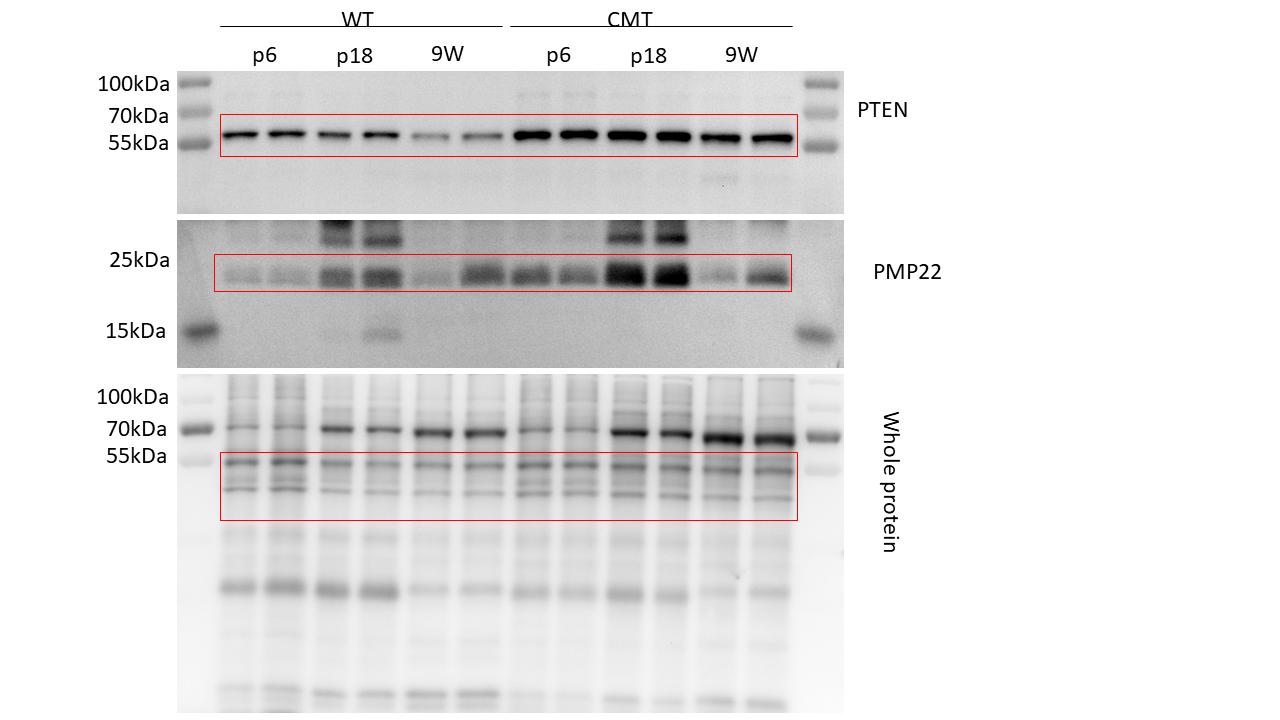

Supplement: Supplementary file 1 — Source Data Fig. 1 [file 44321_2023_19_MOESM1_ESM.zip › Figure1/1C_Blots/timelineCMT1A.tif]

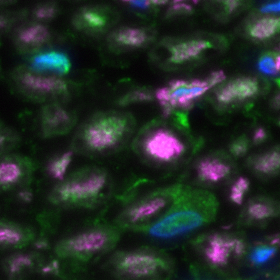

Supplement: Supplementary file 1 — Source Data Fig. 1 [file 44321_2023_19_MOESM1_ESM.zip › Figure1/1H_MicroscopeImages/Compostie_Nfem_CPMP_WT_01.czi - C=0-1.tif]

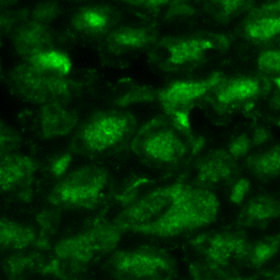

Supplement: Supplementary file 1 — Source Data Fig. 1 [file 44321_2023_19_MOESM1_ESM.zip › Figure1/1H_MicroscopeImages/PTENcst_Nfem_CPMP_WT_01.czi - C=1-1.tif]

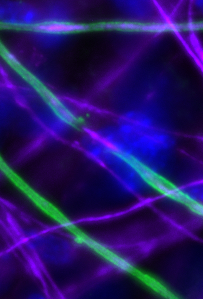

Supplement: Supplementary file 2 — Source Data Fig. 2 [file 44321_2023_19_MOESM2_ESM.zip › Figure2/2B_MicroscopeImages/WT_Ctrl_E740x_small_Composite.tif]

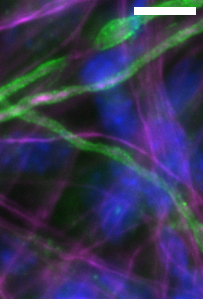

Supplement: Supplementary file 2 — Source Data Fig. 2 [file 44321_2023_19_MOESM2_ESM.zip › Figure2/2B_MicroscopeImages/HNPP_LY10_Composite_10e╠¿m-1.tif]

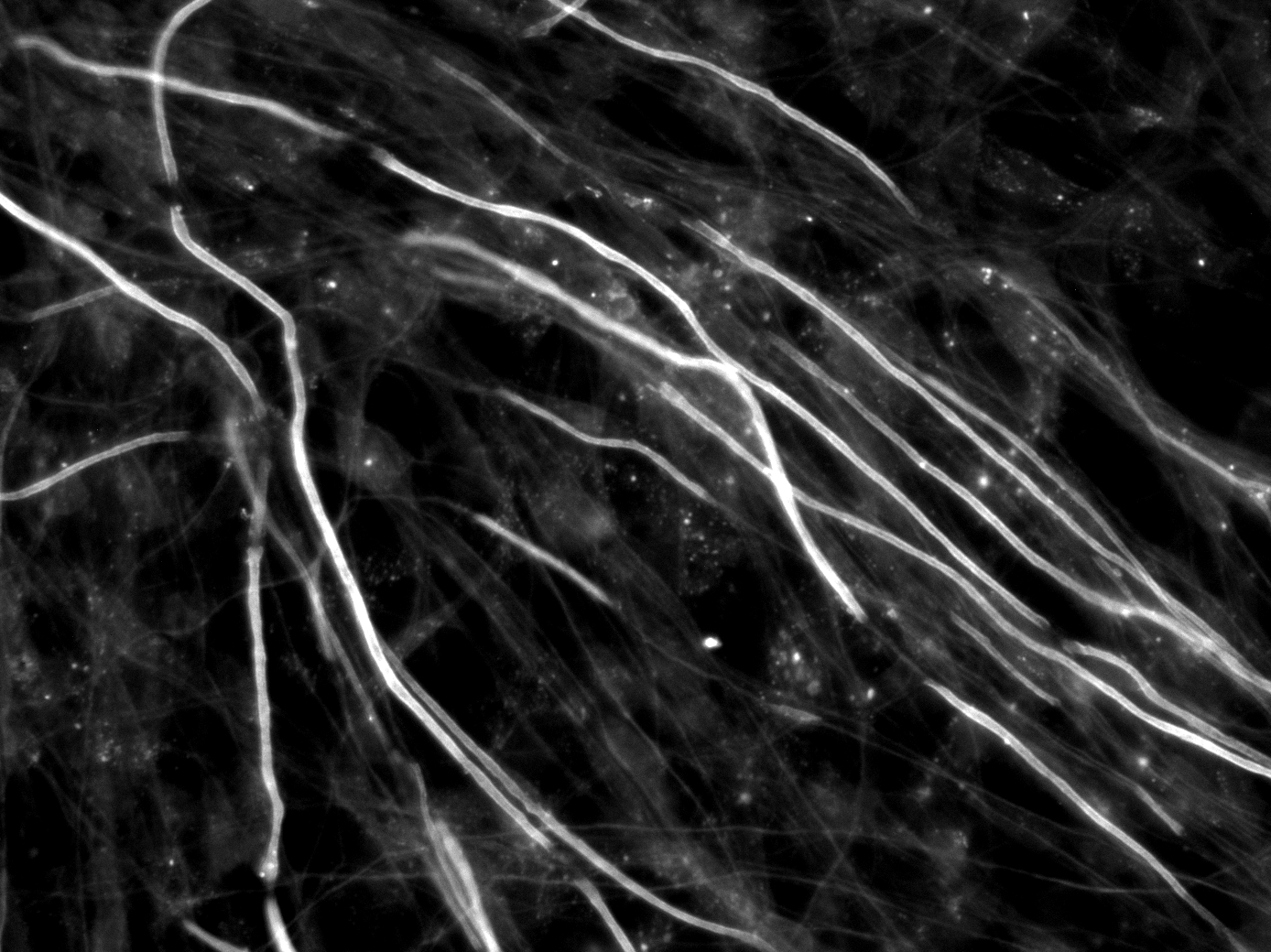

Supplement: Supplementary file 2 — Source Data Fig. 2 [file 44321_2023_19_MOESM2_ESM.zip › Figure2/2B_MicroscopeImages/WT_10e╠¿mLY_MBPgrey_.tif]

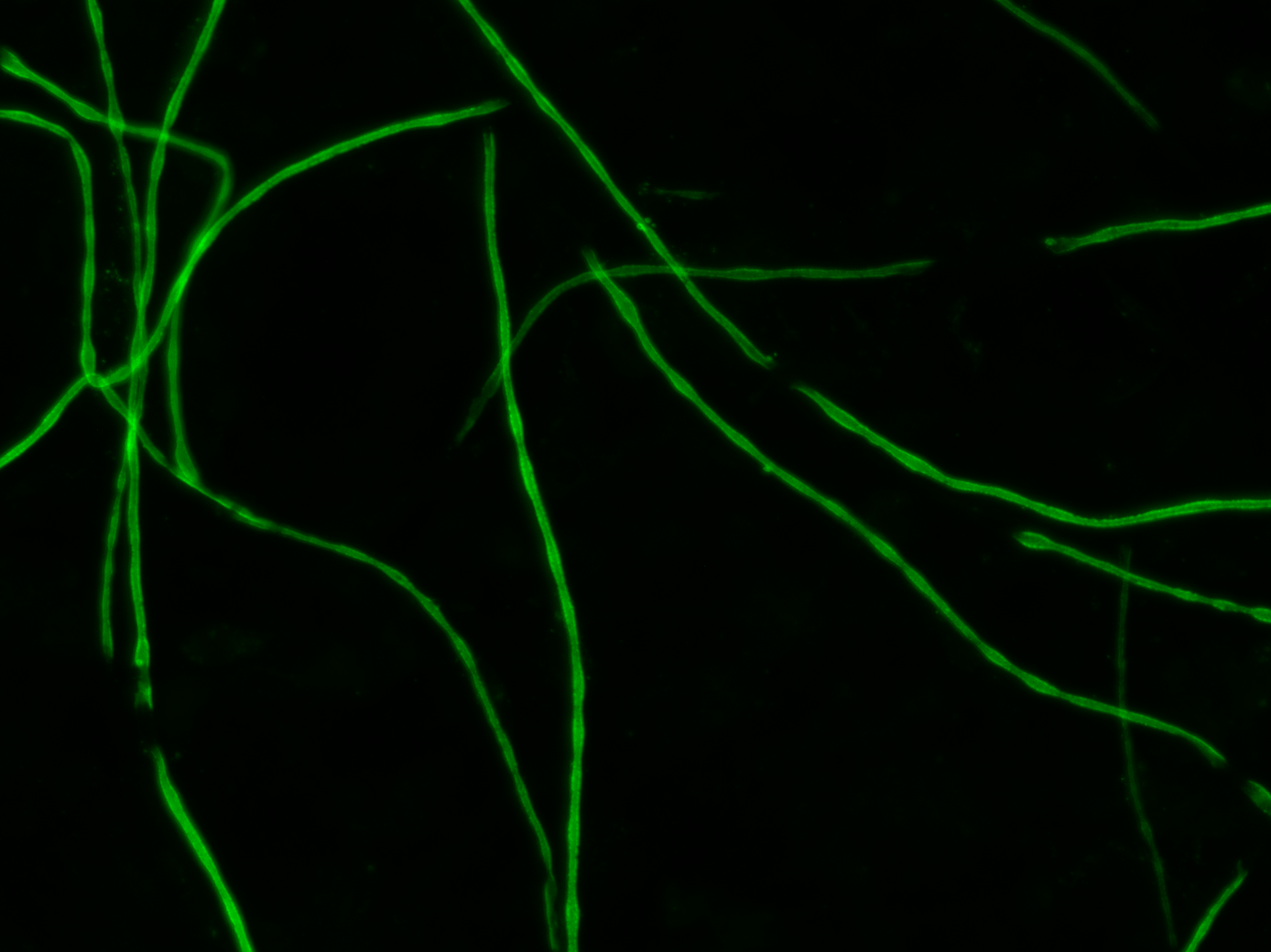

Supplement: Supplementary file 2 — Source Data Fig. 2 [file 44321_2023_19_MOESM2_ESM.zip › Figure2/2B_MicroscopeImages/WT_Ctrl_E740x_MBP.tif]

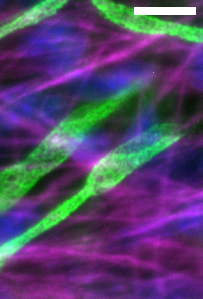

Supplement: Supplementary file 2 — Source Data Fig. 2 [file 44321_2023_19_MOESM2_ESM.zip › Figure2/2B_MicroscopeImages/HNPP_Ctrl_Composite_10e╠¿m-1.tif]

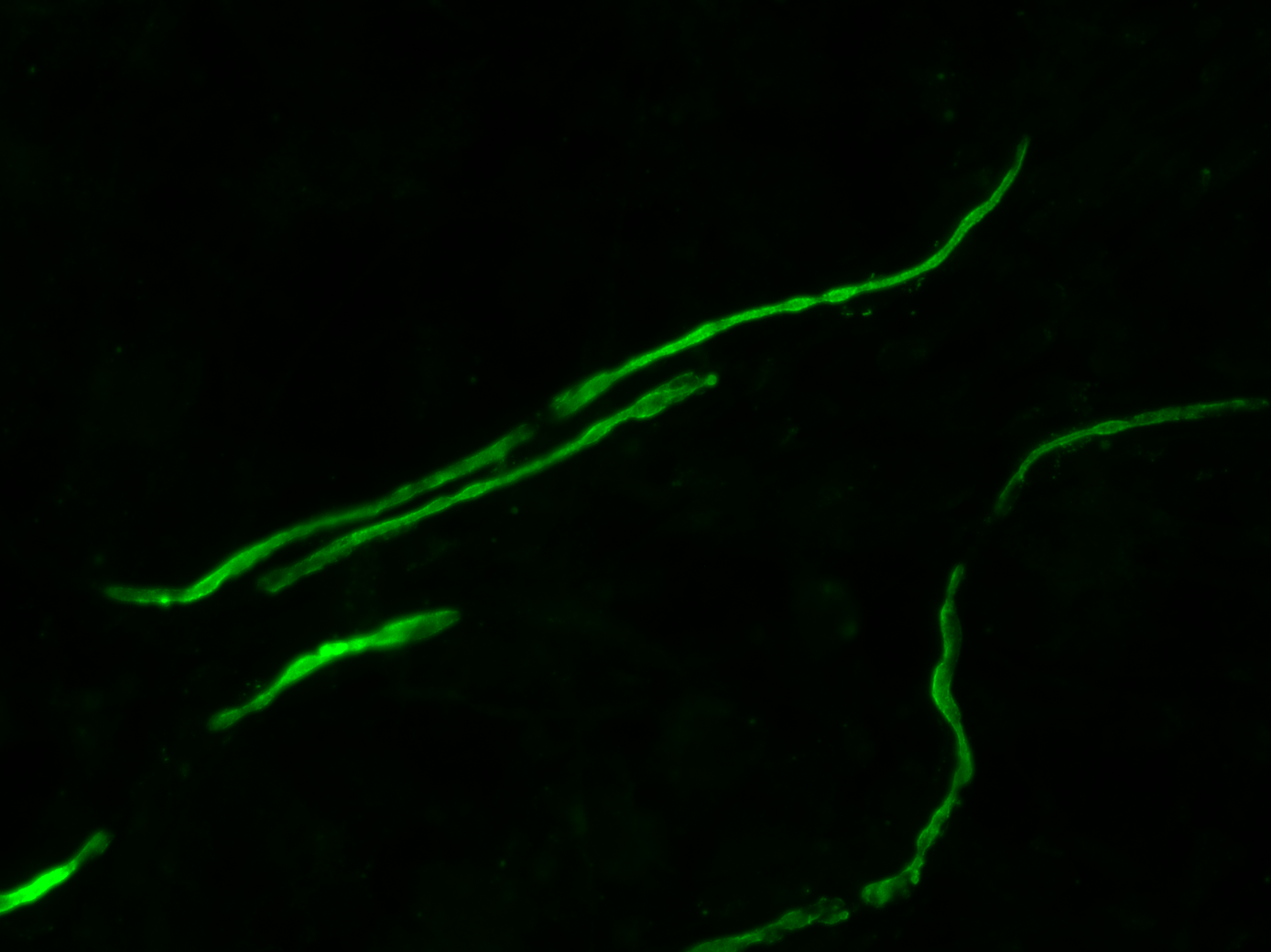

Supplement: Supplementary file 2 — Source Data Fig. 2 [file 44321_2023_19_MOESM2_ESM.zip › Figure2/2B_MicroscopeImages/HNPP_20mMRapa_MBP.tif]

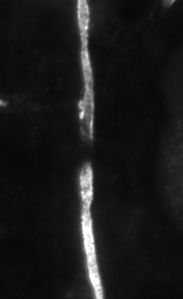

Supplement: Supplementary file 2 — Source Data Fig. 2 [file 44321_2023_19_MOESM2_ESM.zip › Figure2/2B_MicroscopeImages/WT_RAPA_InsetMBP.tif]

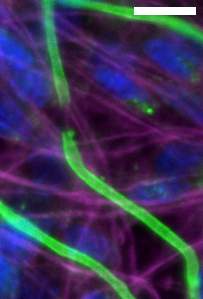

Supplement: Supplementary file 2 — Source Data Fig. 2 [file 44321_2023_19_MOESM2_ESM.zip › Figure2/2B_MicroscopeImages/WT_10e╠¿MLY_Composite_10e╠¿m_inset.tif]

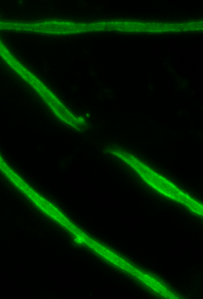

Supplement: Supplementary file 2 — Source Data Fig. 2 [file 44321_2023_19_MOESM2_ESM.zip › Figure2/2B_MicroscopeImages/WT_Ctrl_E740x_small_MBP.tif]

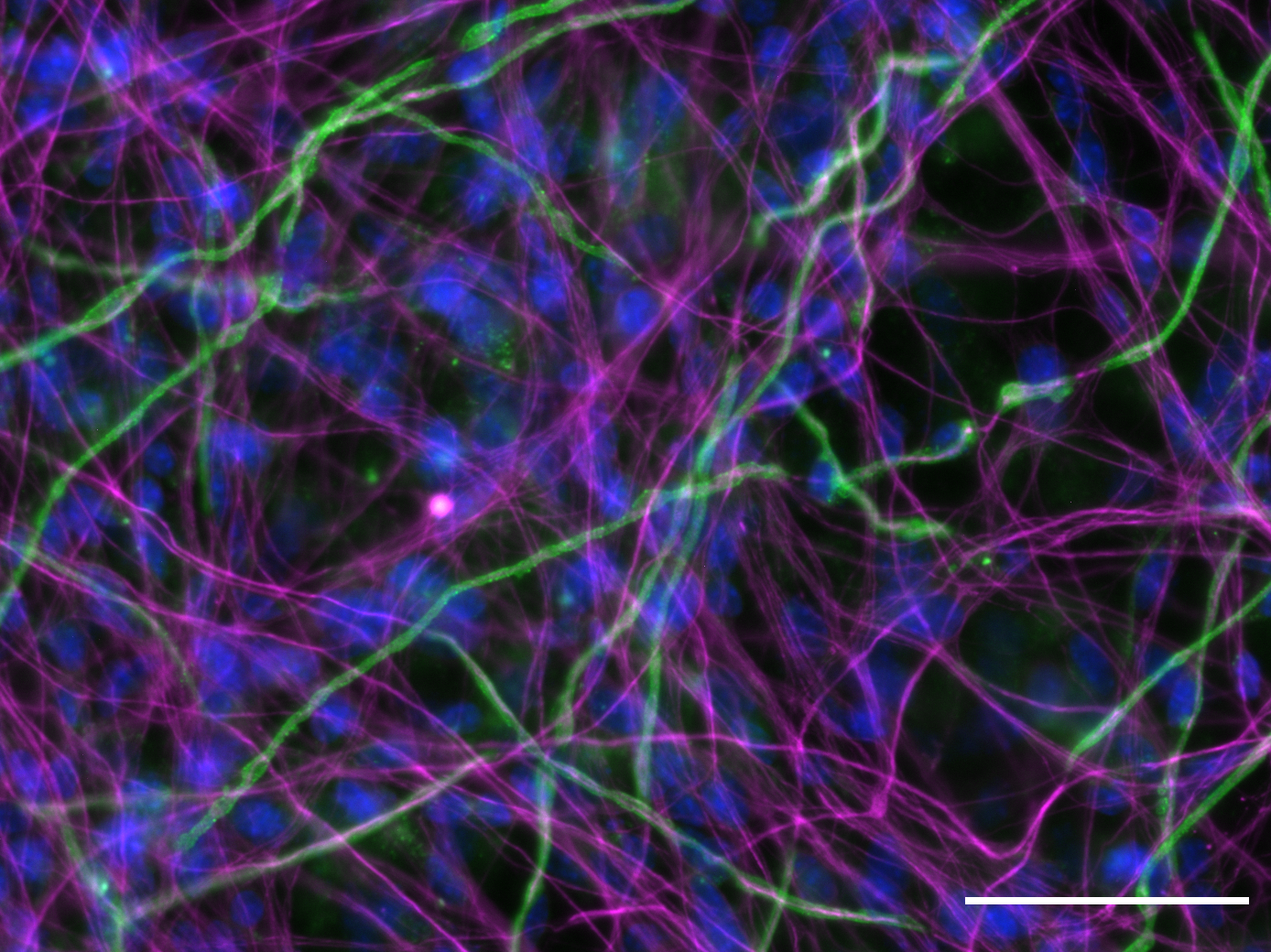

Supplement: Supplementary file 2 — Source Data Fig. 2 [file 44321_2023_19_MOESM2_ESM.zip › Figure2/2B_MicroscopeImages/HNPP_LY10_Composite_50e╠¿m.tif]

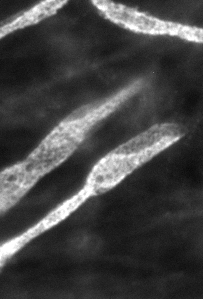

Supplement: Supplementary file 2 — Source Data Fig. 2 [file 44321_2023_19_MOESM2_ESM.zip › Figure2/2B_MicroscopeImages/HNPP_Ctrl_Greys_inset.tif]

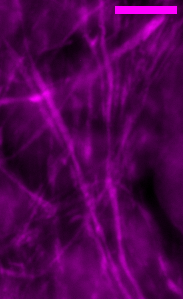

Supplement: Supplementary file 2 — Source Data Fig. 2 [file 44321_2023_19_MOESM2_ESM.zip › Figure2/2B_MicroscopeImages/WT_RAPA_InsetTUJ.tif]

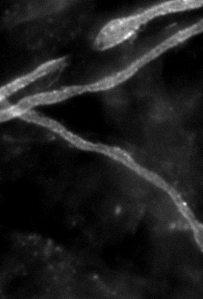

Supplement: Supplementary file 2 — Source Data Fig. 2 [file 44321_2023_19_MOESM2_ESM.zip › Figure2/2B_MicroscopeImages/HNPP_LY10_Greys_inset.tif]

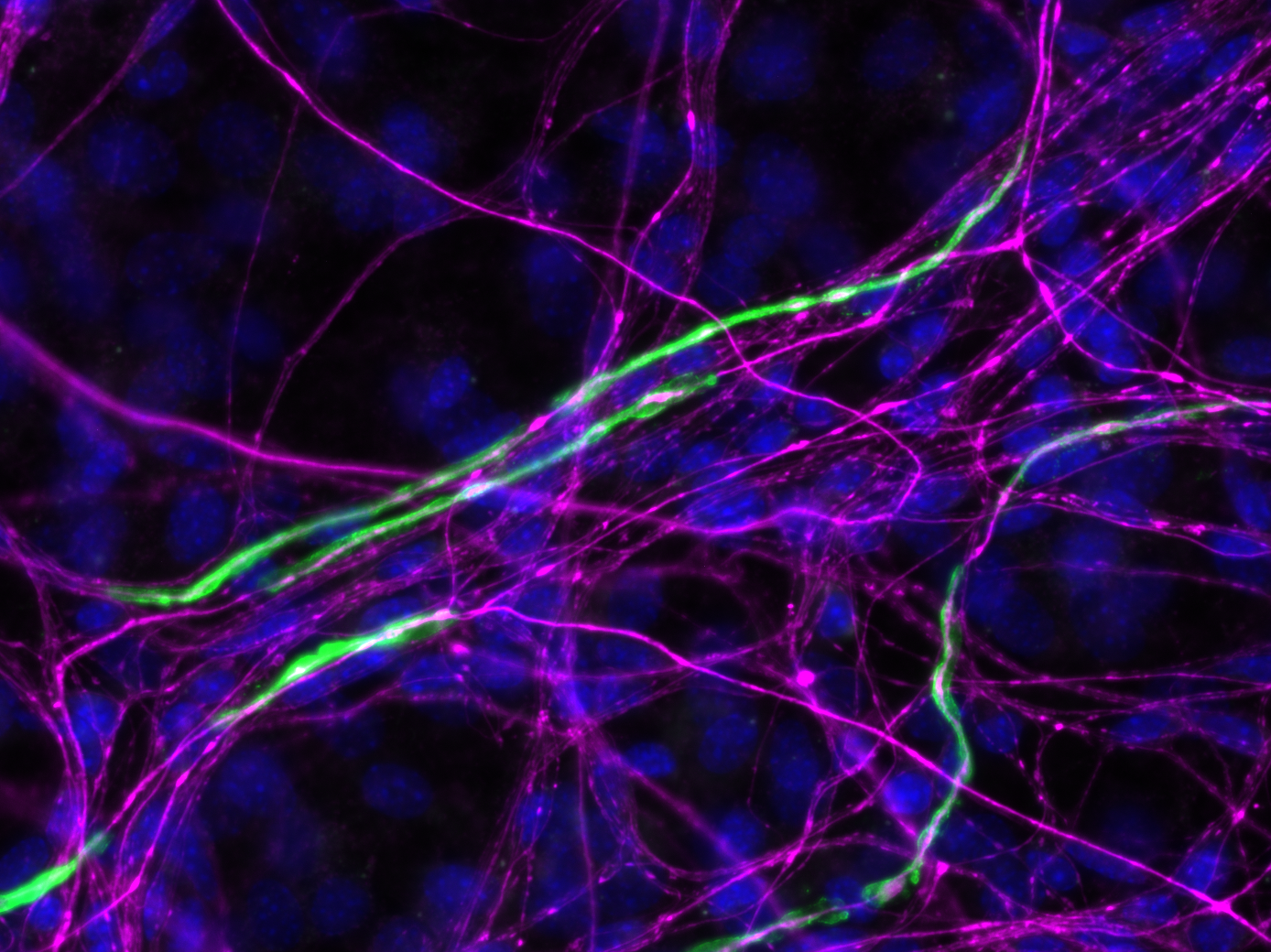

Supplement: Supplementary file 2 — Source Data Fig. 2 [file 44321_2023_19_MOESM2_ESM.zip › Figure2/2B_MicroscopeImages/HNPP_20mMRapa_Composite.tif]

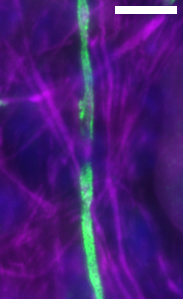

Supplement: Supplementary file 2 — Source Data Fig. 2 [file 44321_2023_19_MOESM2_ESM.zip › Figure2/2B_MicroscopeImages/WT_RAPA_InsetComposite10mm.tif]

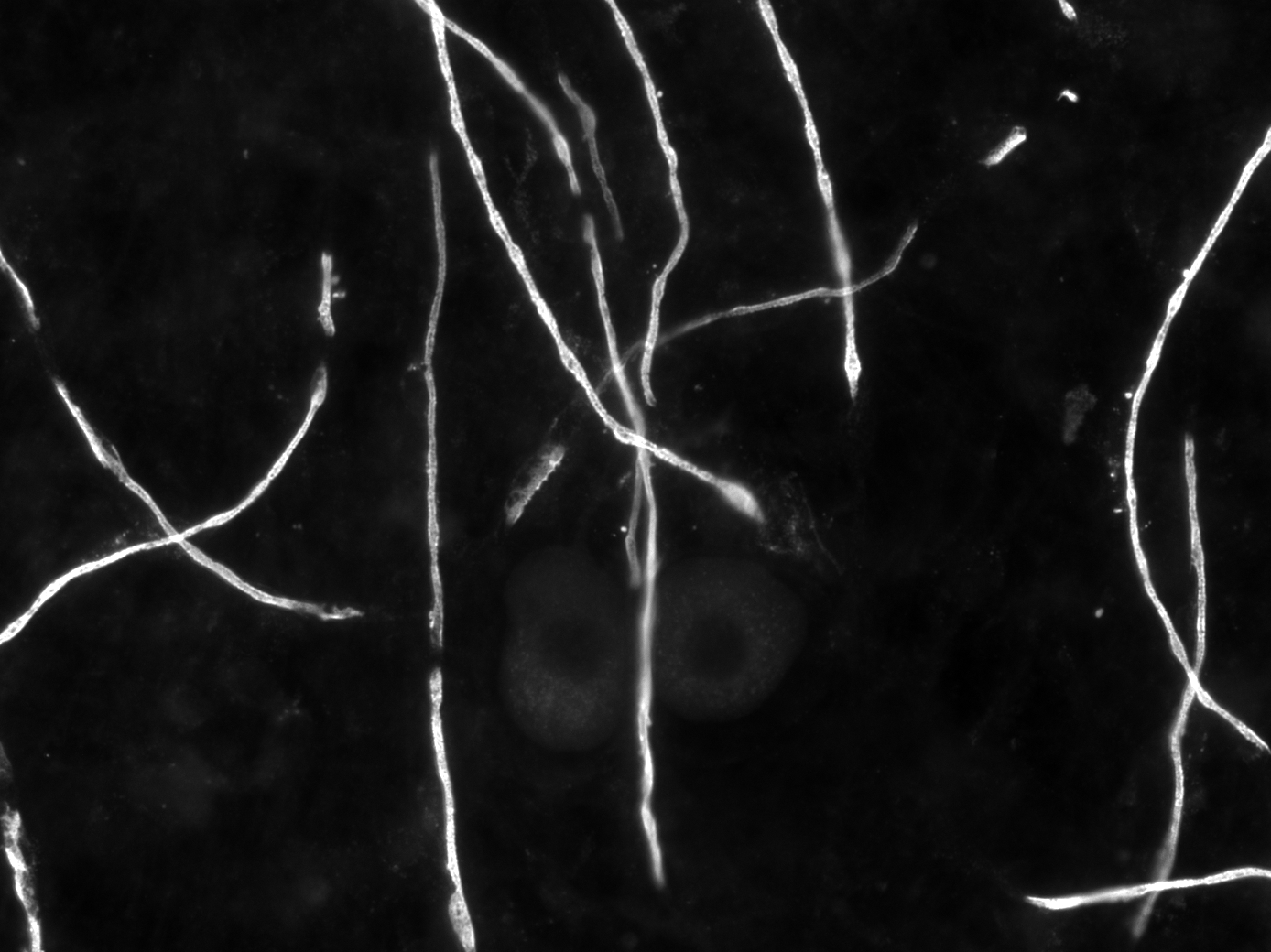

Supplement: Supplementary file 2 — Source Data Fig. 2 [file 44321_2023_19_MOESM2_ESM.zip › Figure2/2B_MicroscopeImages/WT_RAPA_MBPgrey.tif]

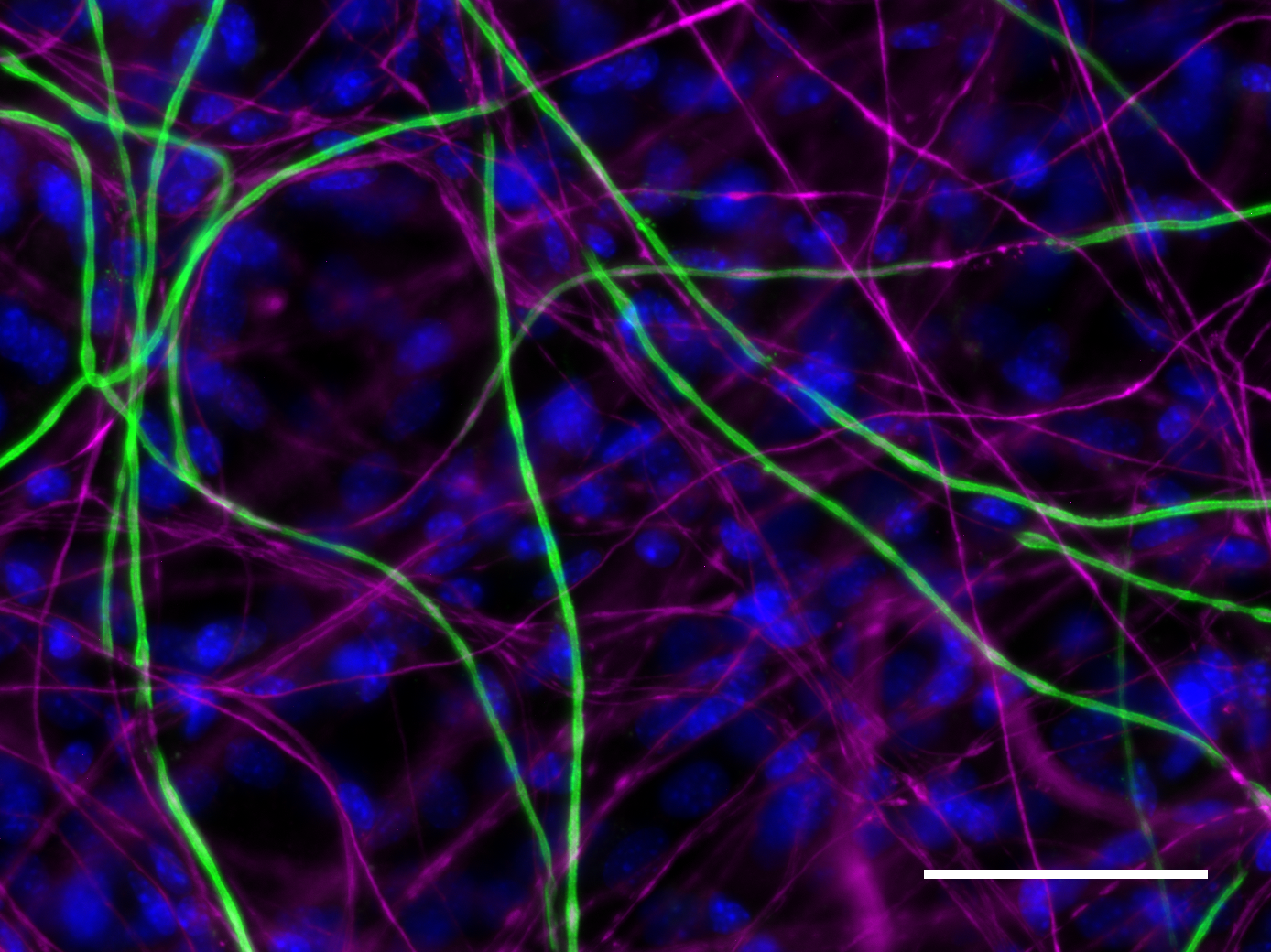

Supplement: Supplementary file 2 — Source Data Fig. 2 [file 44321_2023_19_MOESM2_ESM.zip › Figure2/2B_MicroscopeImages/WT_Ctrl_E740x_Composite_50e╠¿m.tif]

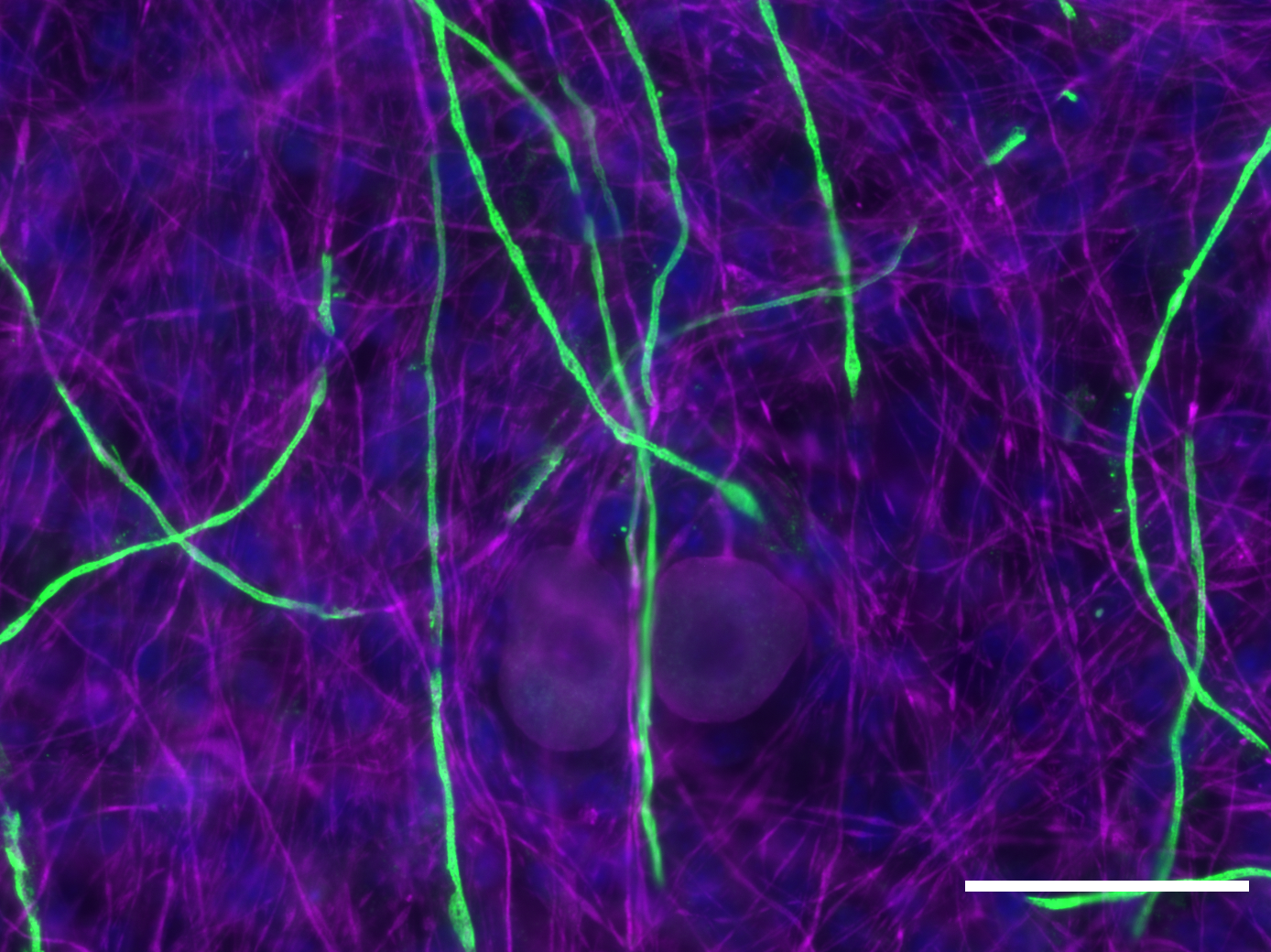

Supplement: Supplementary file 2 — Source Data Fig. 2 [file 44321_2023_19_MOESM2_ESM.zip › Figure2/2B_MicroscopeImages/WT_RAPA_Composite50e╠¿m.tif]

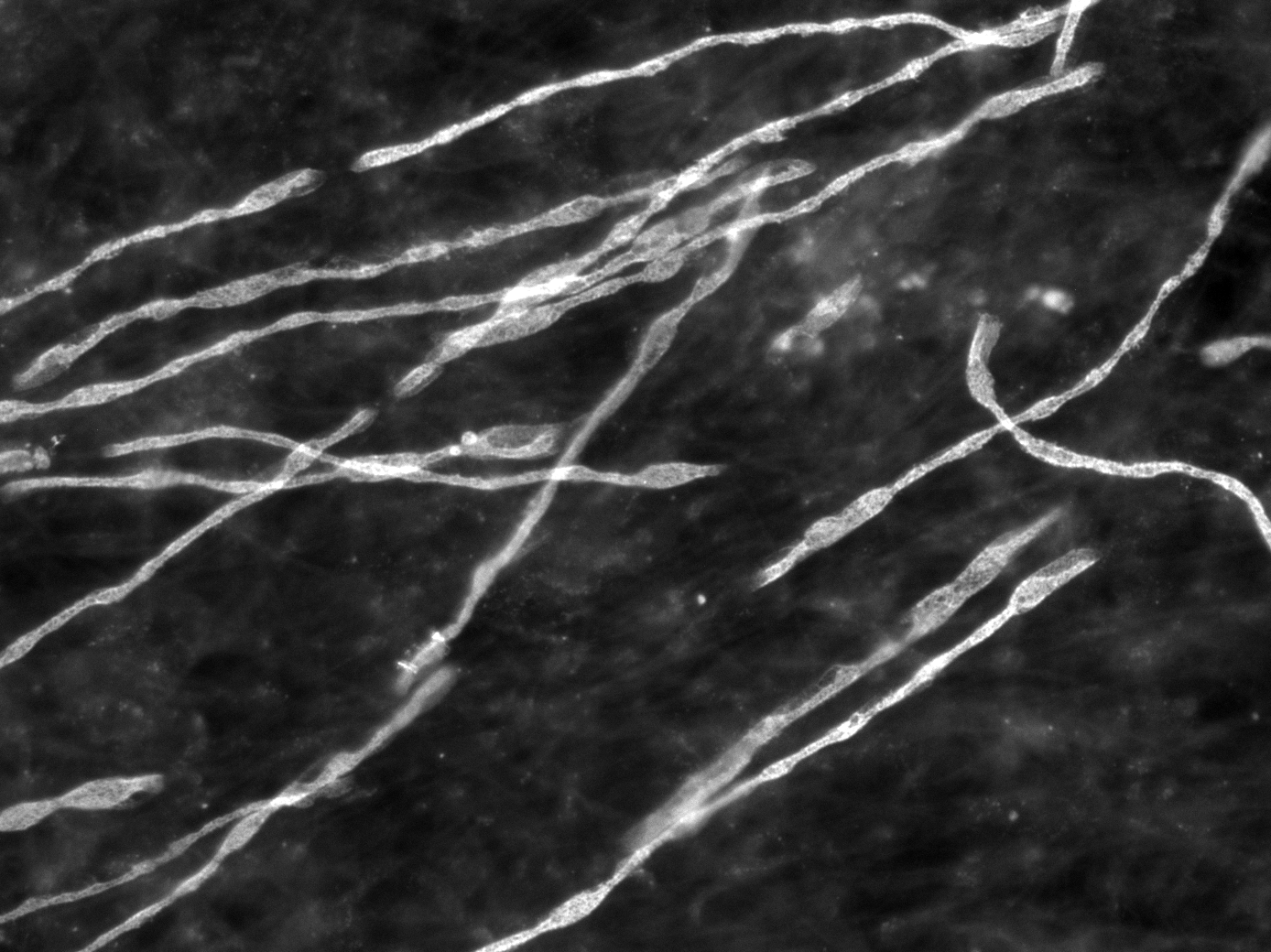

Supplement: Supplementary file 2 — Source Data Fig. 2 [file 44321_2023_19_MOESM2_ESM.zip › Figure2/2B_MicroscopeImages/HNPP_Ctrl_Greys_50e╠¿m.tif]

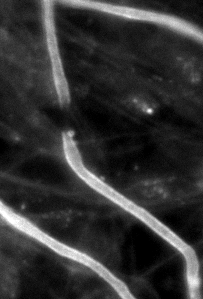

Supplement: Supplementary file 2 — Source Data Fig. 2 [file 44321_2023_19_MOESM2_ESM.zip › Figure2/2B_MicroscopeImages/WT_10e╠¿mLY_MBPgrey_inset_.tif]

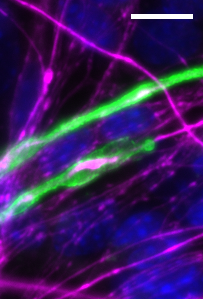

Supplement: Supplementary file 2 — Source Data Fig. 2 [file 44321_2023_19_MOESM2_ESM.zip › Figure2/2B_MicroscopeImages/HNPP_20mMRapa_small_Composite_10e╠¿m.tif]

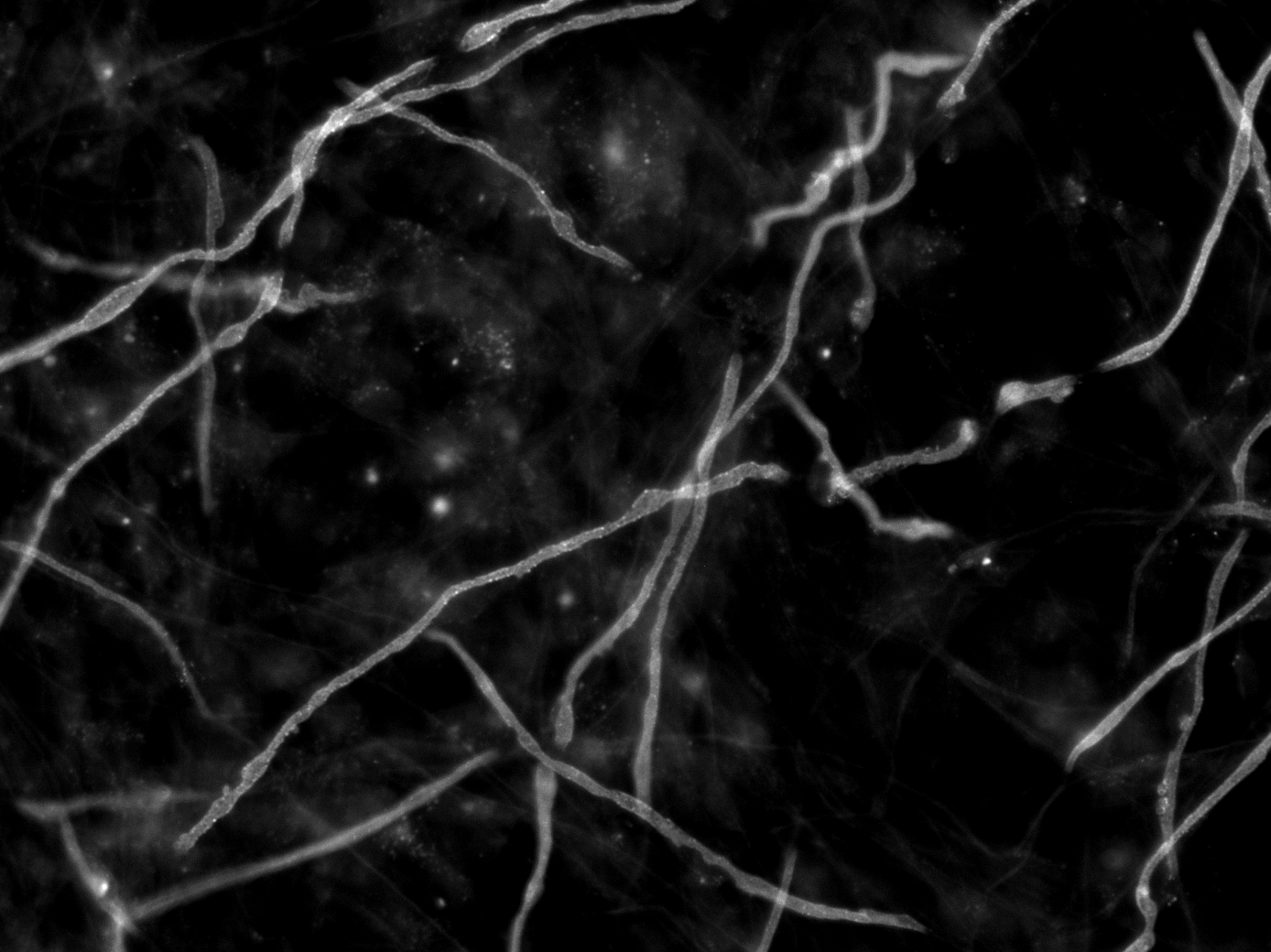

Supplement: Supplementary file 2 — Source Data Fig. 2 [file 44321_2023_19_MOESM2_ESM.zip › Figure2/2B_MicroscopeImages/HNPP_LY10_Greys_50e╠¿m.tif]

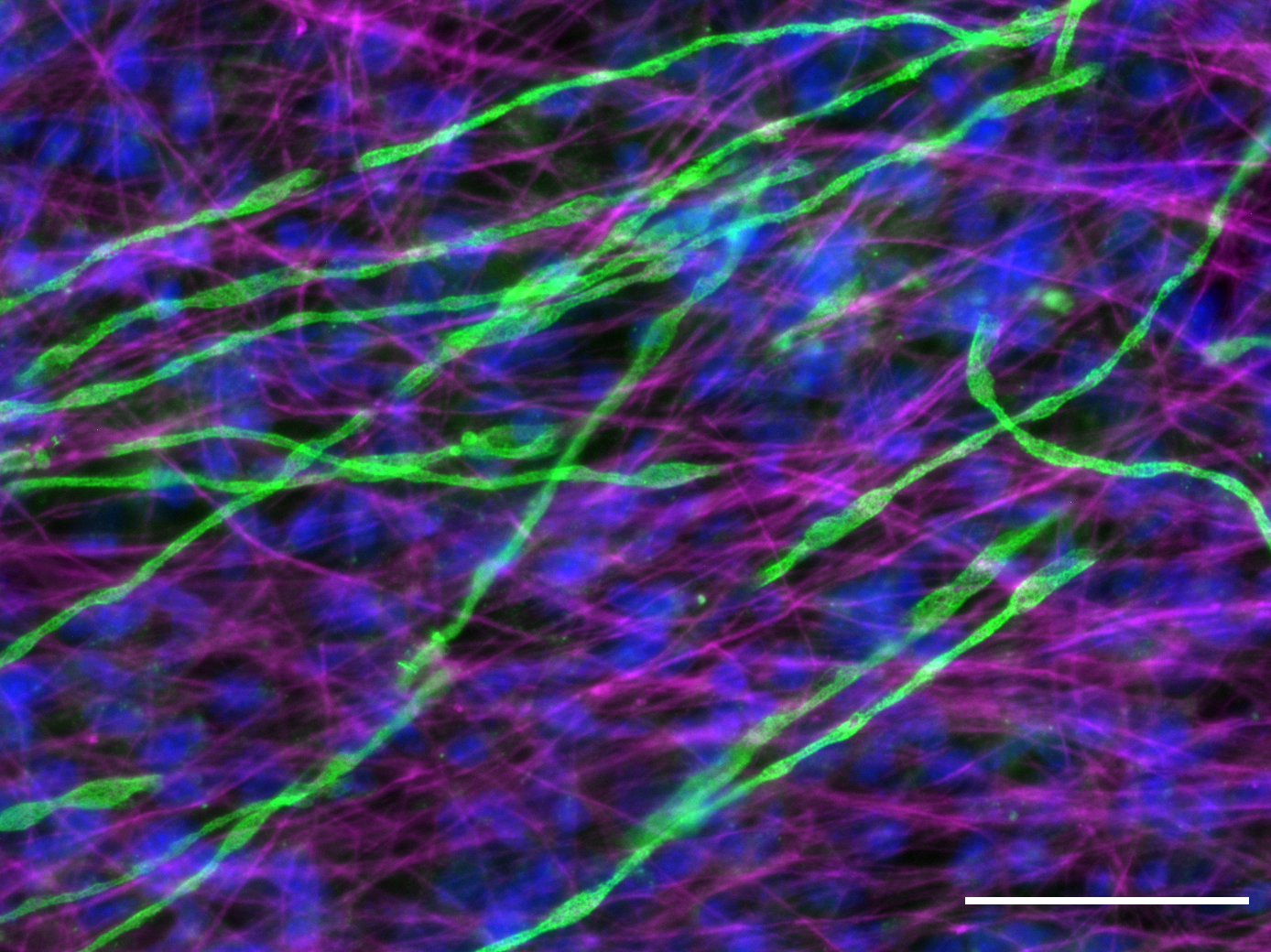

Supplement: Supplementary file 2 — Source Data Fig. 2 [file 44321_2023_19_MOESM2_ESM.zip › Figure2/2B_MicroscopeImages/HNPP_Ctrl_Composite_50e╠¿m.tif]

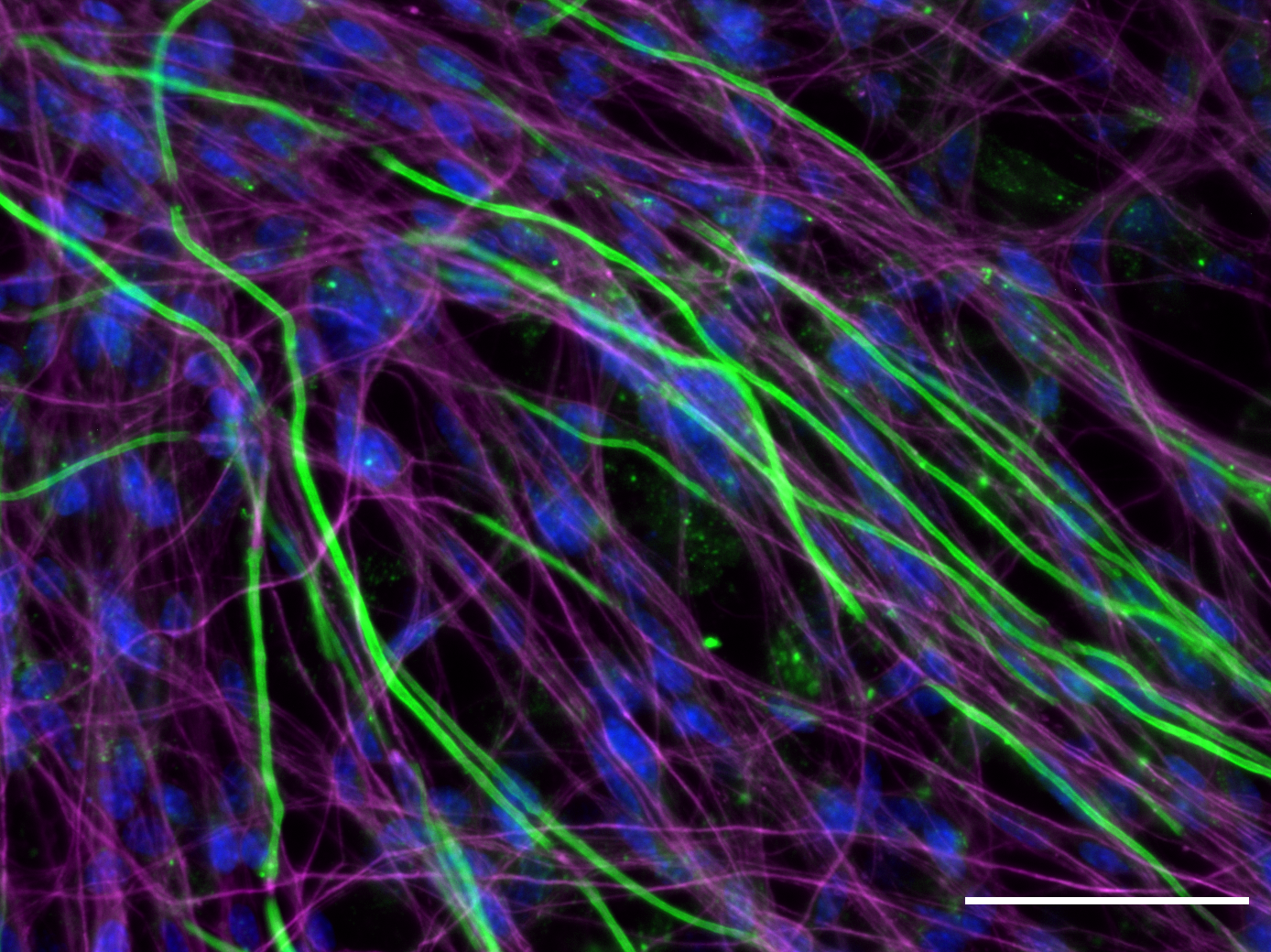

Supplement: Supplementary file 2 — Source Data Fig. 2 [file 44321_2023_19_MOESM2_ESM.zip › Figure2/2B_MicroscopeImages/WT_10e╠¿mLY_Composite_50e╠¿m.tif]

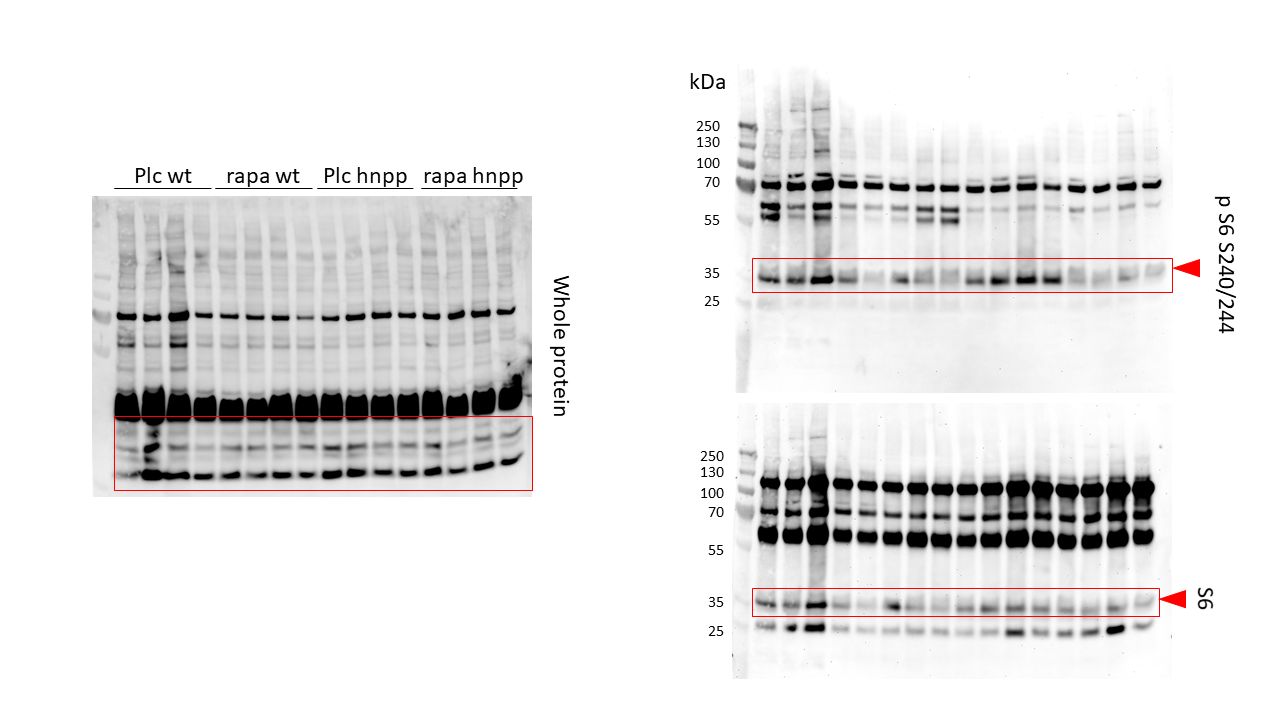

Supplement: Supplementary file 3 — Source Data Fig. 3 [file 44321_2023_19_MOESM3_ESM.zip › Figure3/3B_Blots/3B_PS6-S6Blot.tif]

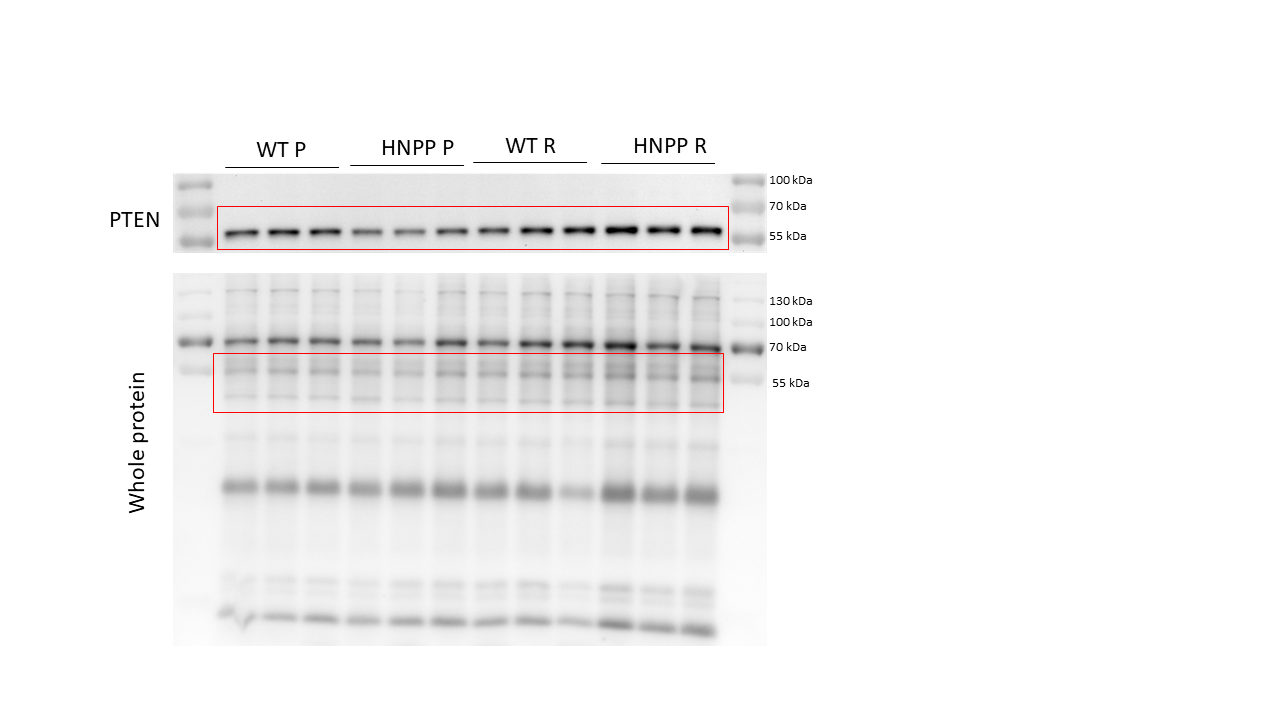

Supplement: Supplementary file 3 — Source Data Fig. 3 [file 44321_2023_19_MOESM3_ESM.zip › Figure3/3B_Blots/3B_PTENBlot.tif]

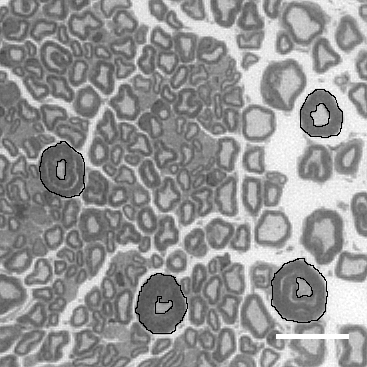

Supplement: Supplementary file 3 — Source Data Fig. 3 [file 44321_2023_19_MOESM3_ESM.zip › Figure3/3C_Images/_BspBildHNPP_Rapa_20e╠¿m.tif]

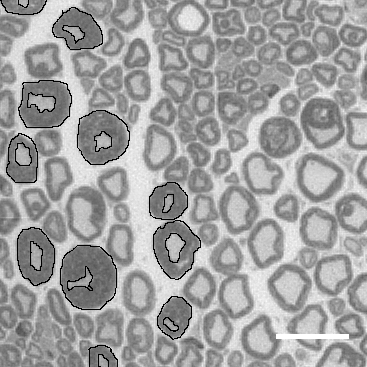

Supplement: Supplementary file 3 — Source Data Fig. 3 [file 44321_2023_19_MOESM3_ESM.zip › Figure3/3C_Images/_BspBildHNPP_Placebo_20e╠¿m.tif]

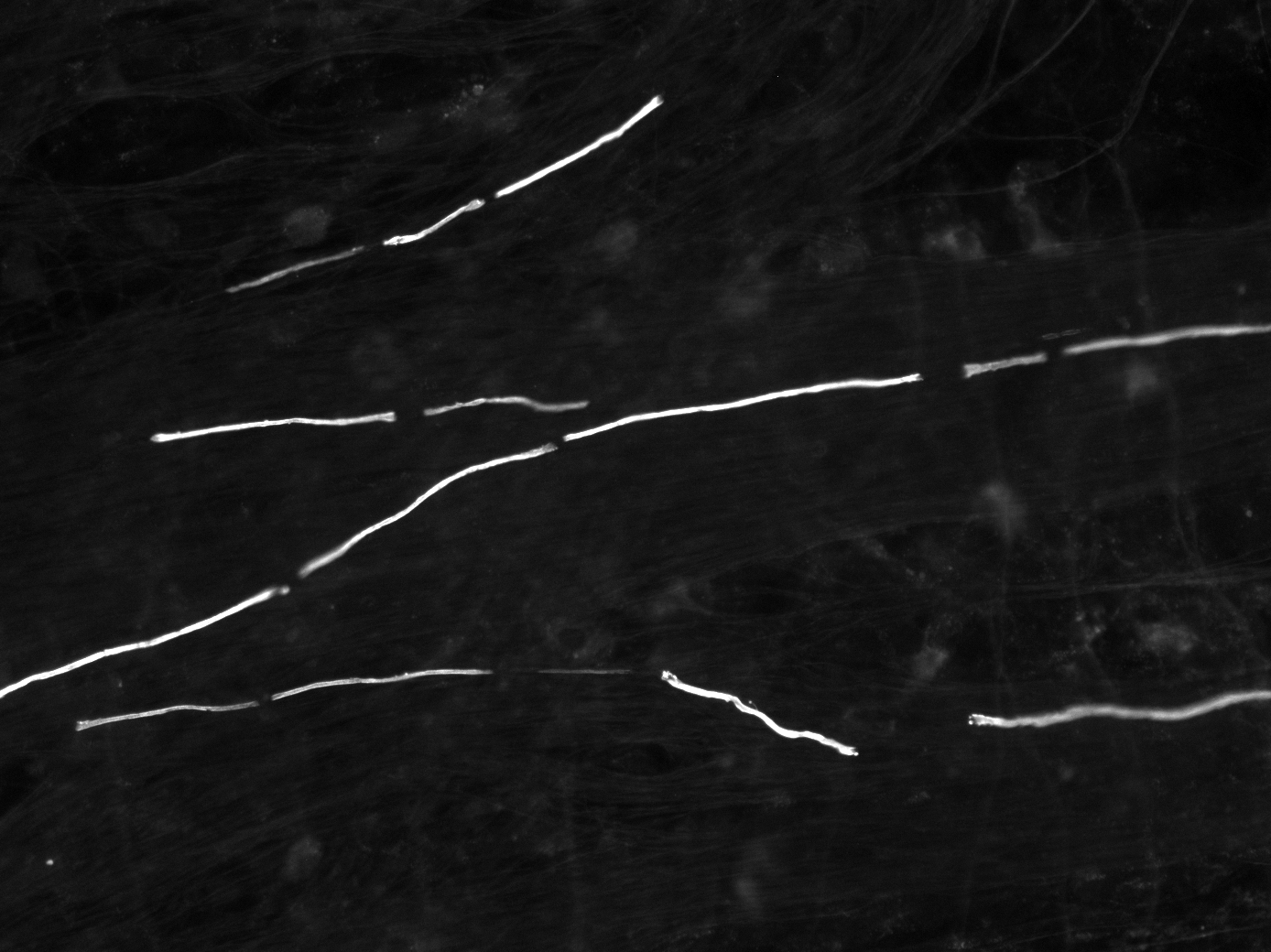

Supplement: Supplementary file 4 — Source Data Fig. 4 [file 44321_2023_19_MOESM4_ESM.zip › Figure4/4B_Images/WT_500_MBP_.tif]

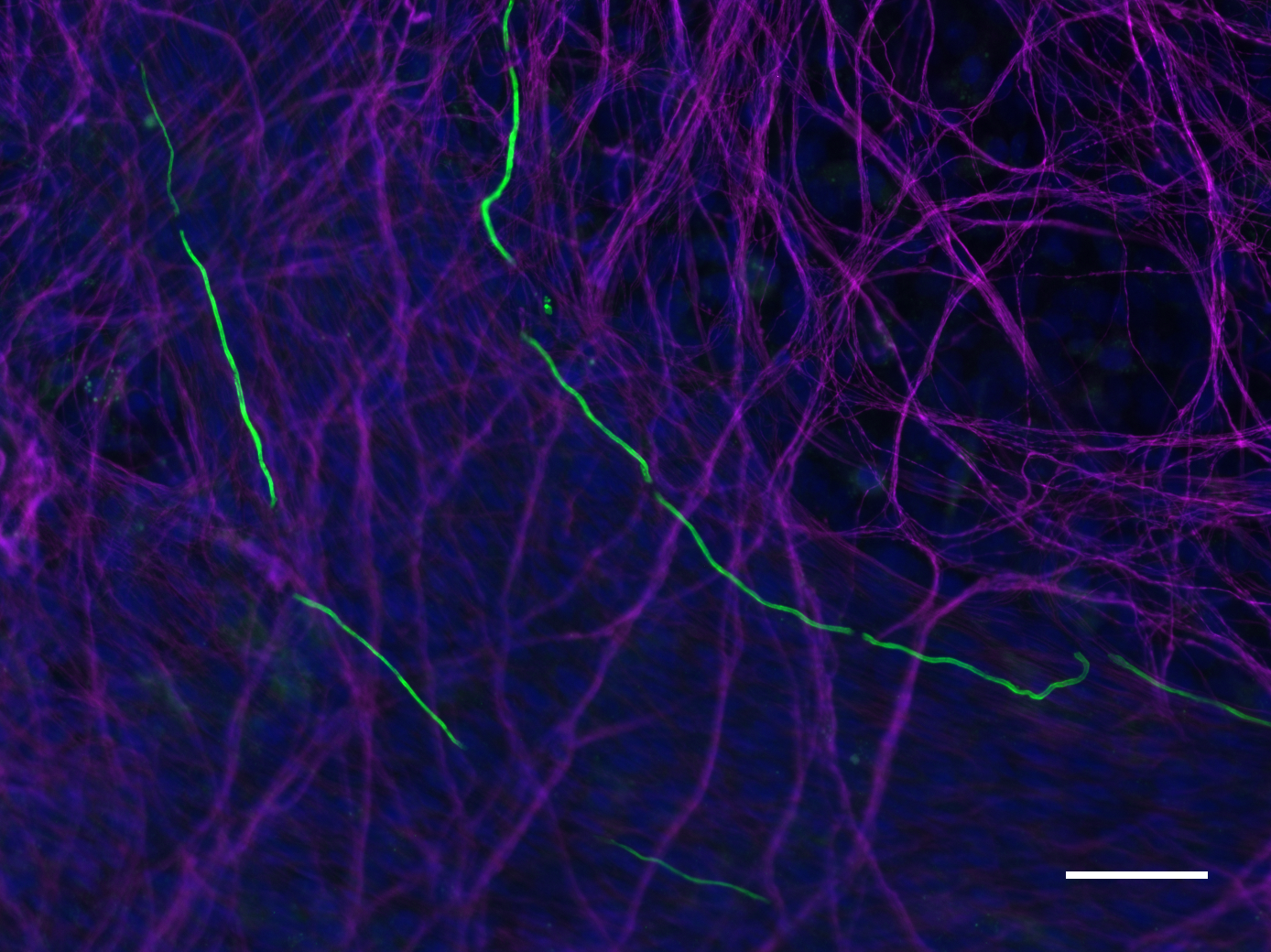

Supplement: Supplementary file 4 — Source Data Fig. 4 [file 44321_2023_19_MOESM4_ESM.zip › Figure4/4B_Images/CMT_500_Composite_50e╠¿m.tif]

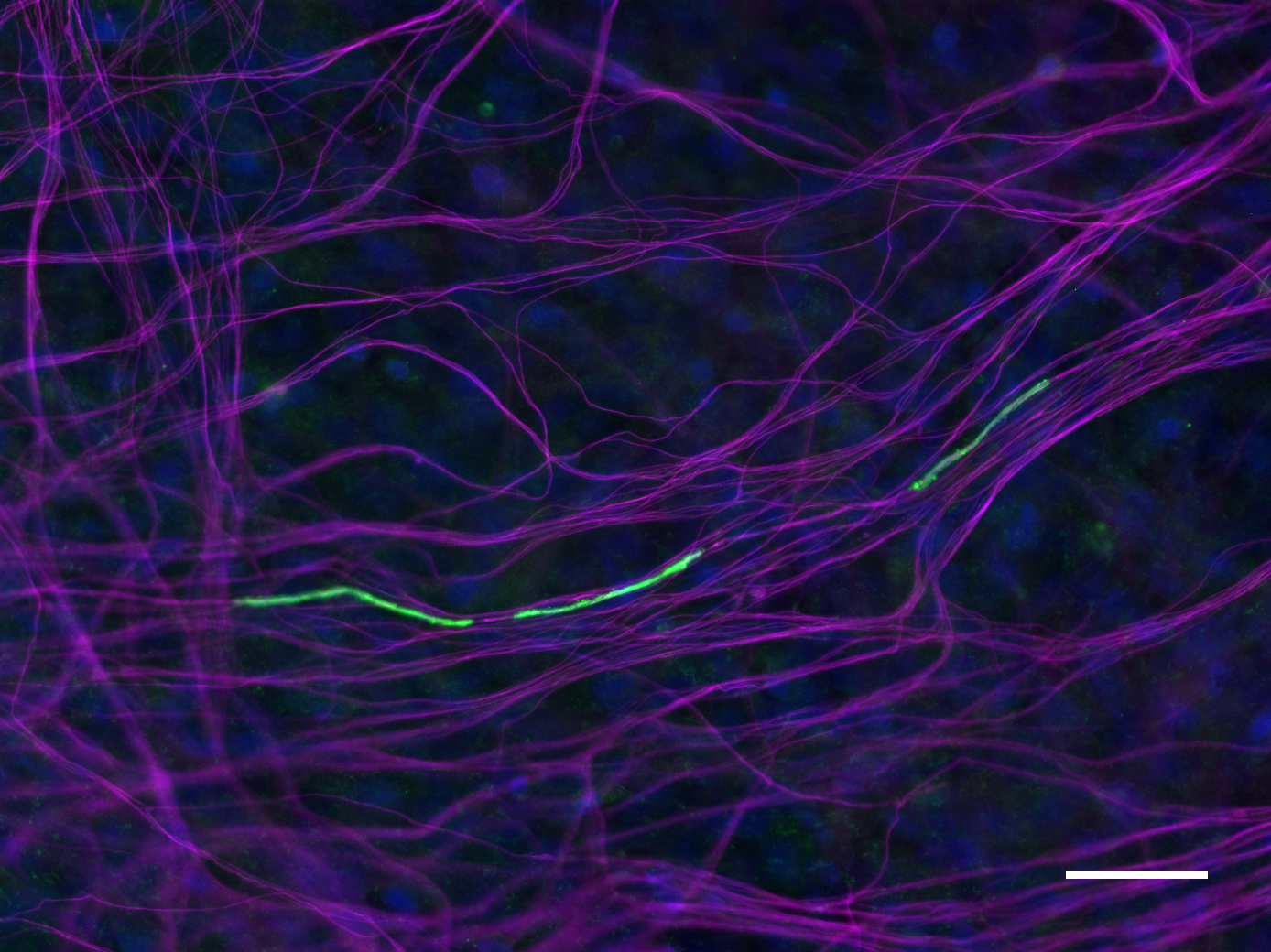

Supplement: Supplementary file 4 — Source Data Fig. 4 [file 44321_2023_19_MOESM4_ESM.zip › Figure4/4B_Images/CMT_CTRL_Composite_50e╠¿m.tif]

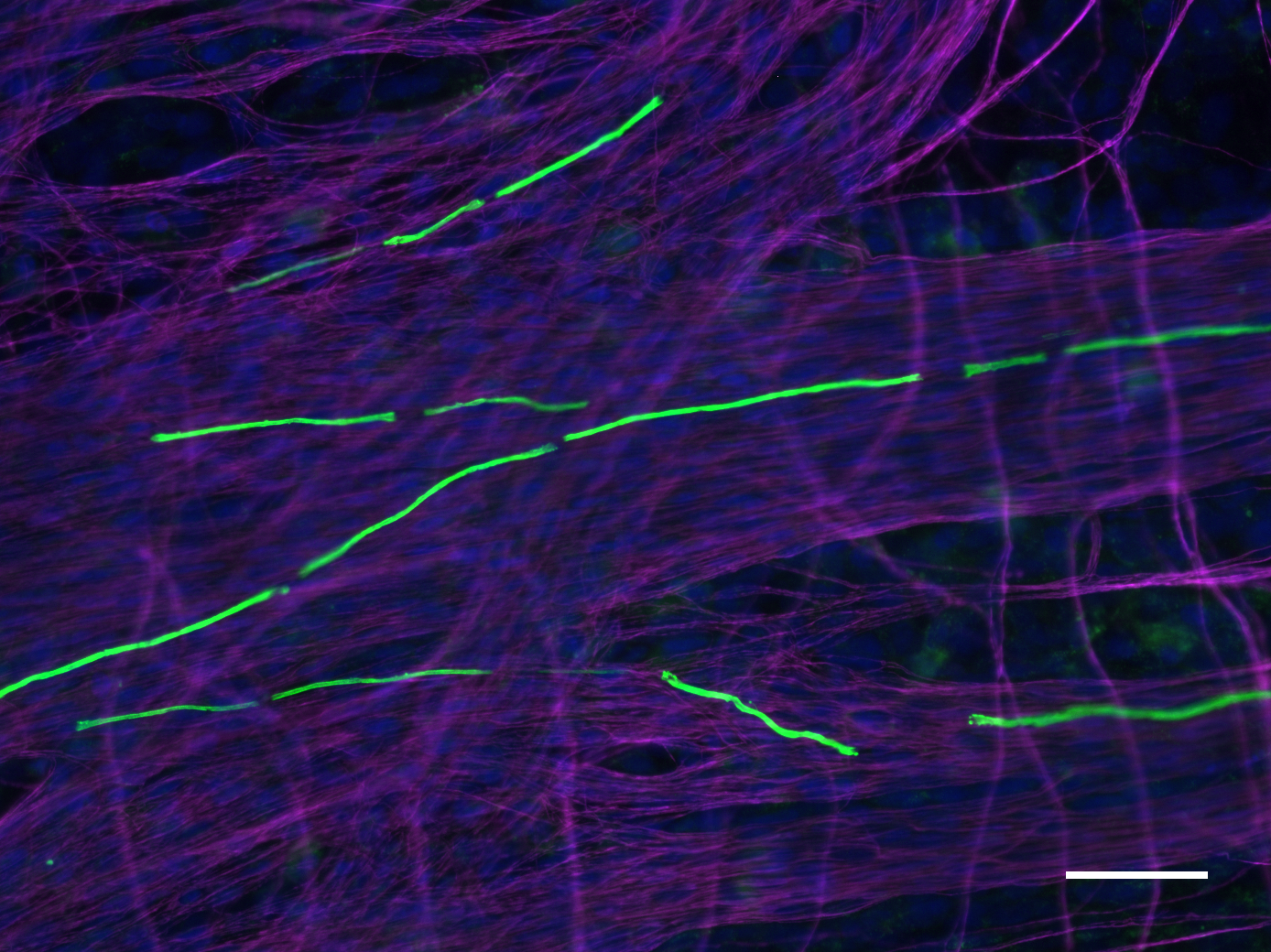

Supplement: Supplementary file 4 — Source Data Fig. 4 [file 44321_2023_19_MOESM4_ESM.zip › Figure4/4B_Images/WT_500_Composite_50e╠¿m.tif]

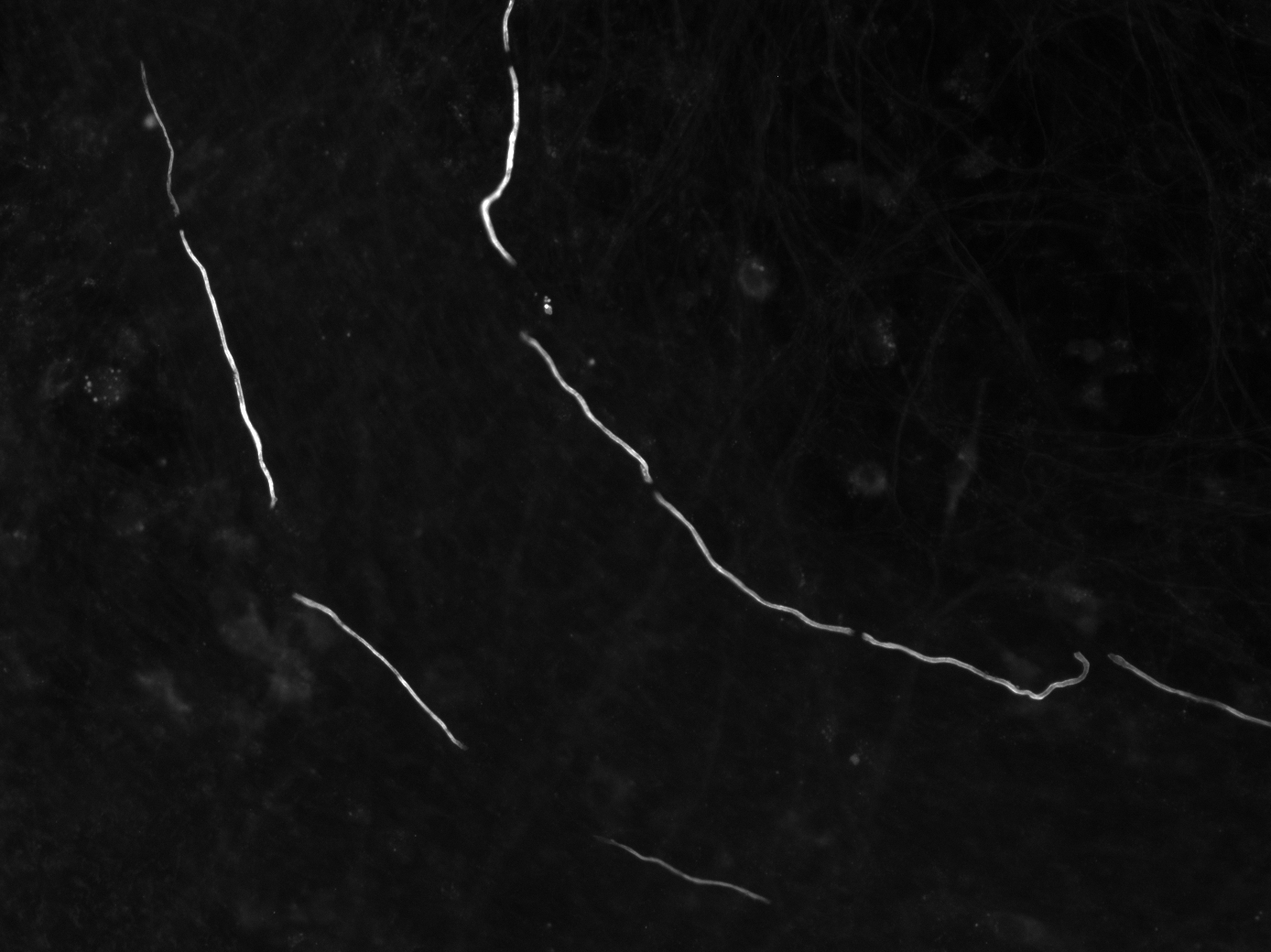

Supplement: Supplementary file 4 — Source Data Fig. 4 [file 44321_2023_19_MOESM4_ESM.zip › Figure4/4B_Images/CMT_500_MBP_.tif]

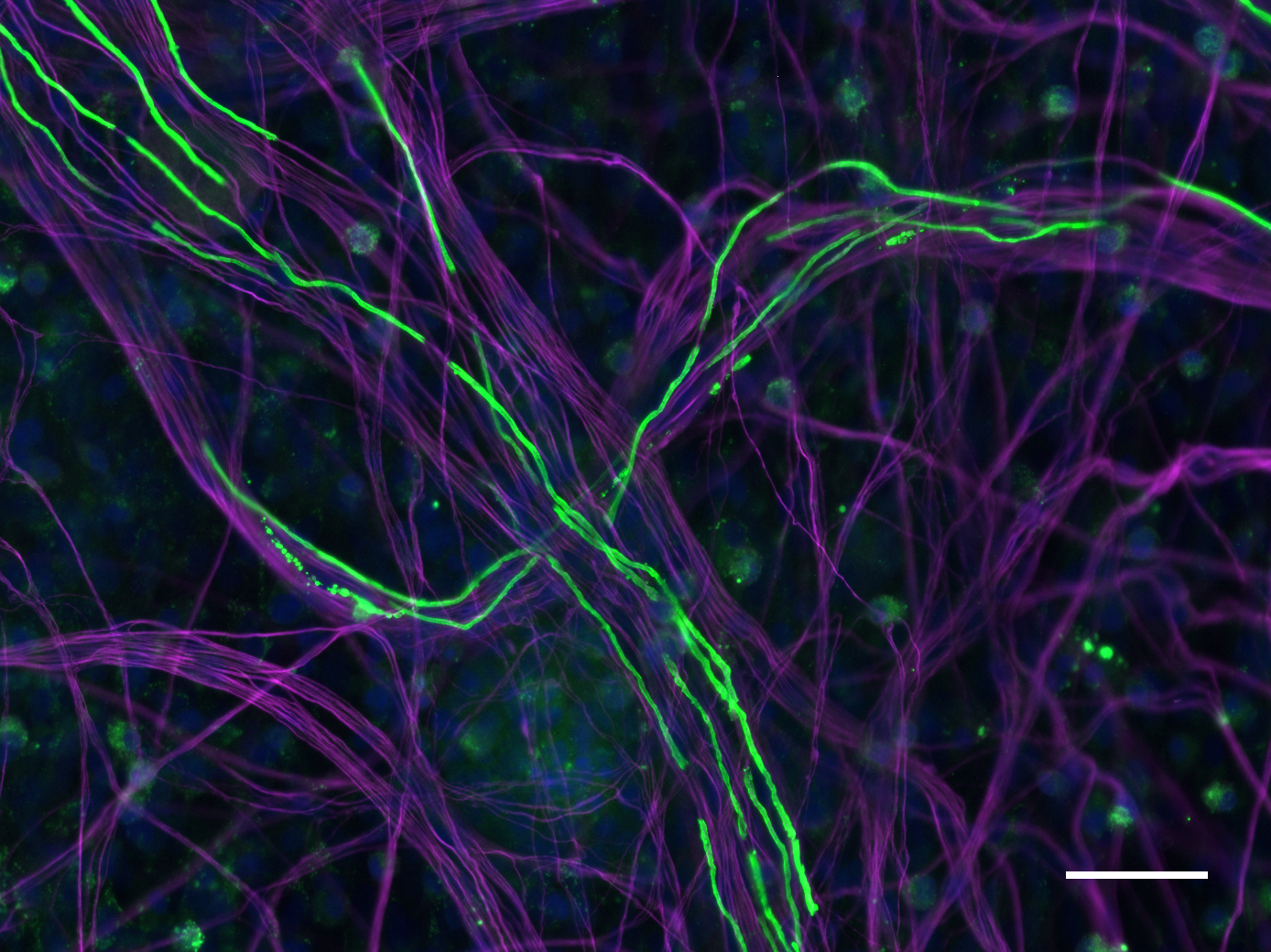

Supplement: Supplementary file 4 — Source Data Fig. 4 [file 44321_2023_19_MOESM4_ESM.zip › Figure4/4B_Images/WT_Ctrl_Composite_50e╠¿m.tif]

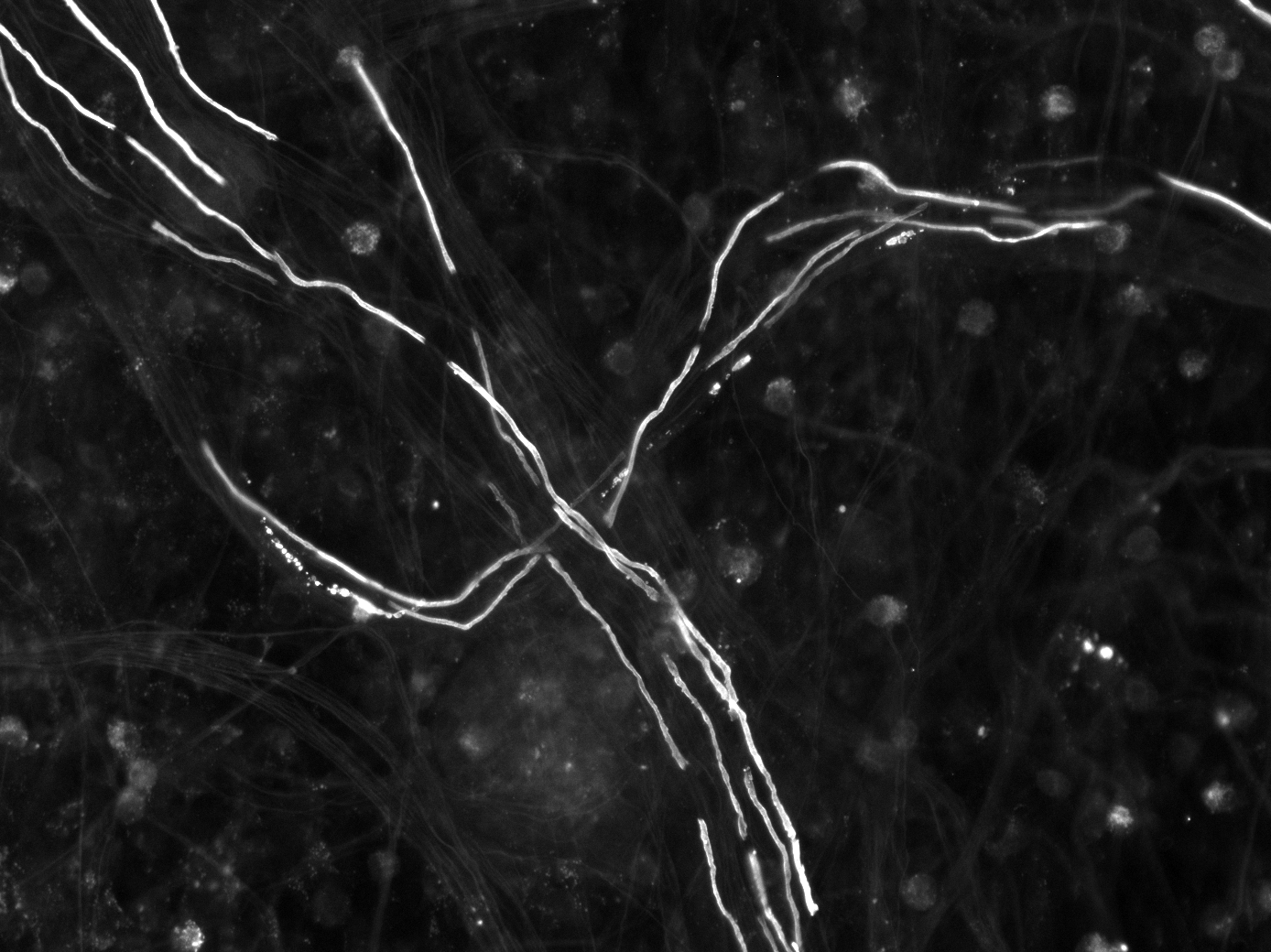

Supplement: Supplementary file 4 — Source Data Fig. 4 [file 44321_2023_19_MOESM4_ESM.zip › Figure4/4B_Images/WT_Ctrl_MBP_.tif]

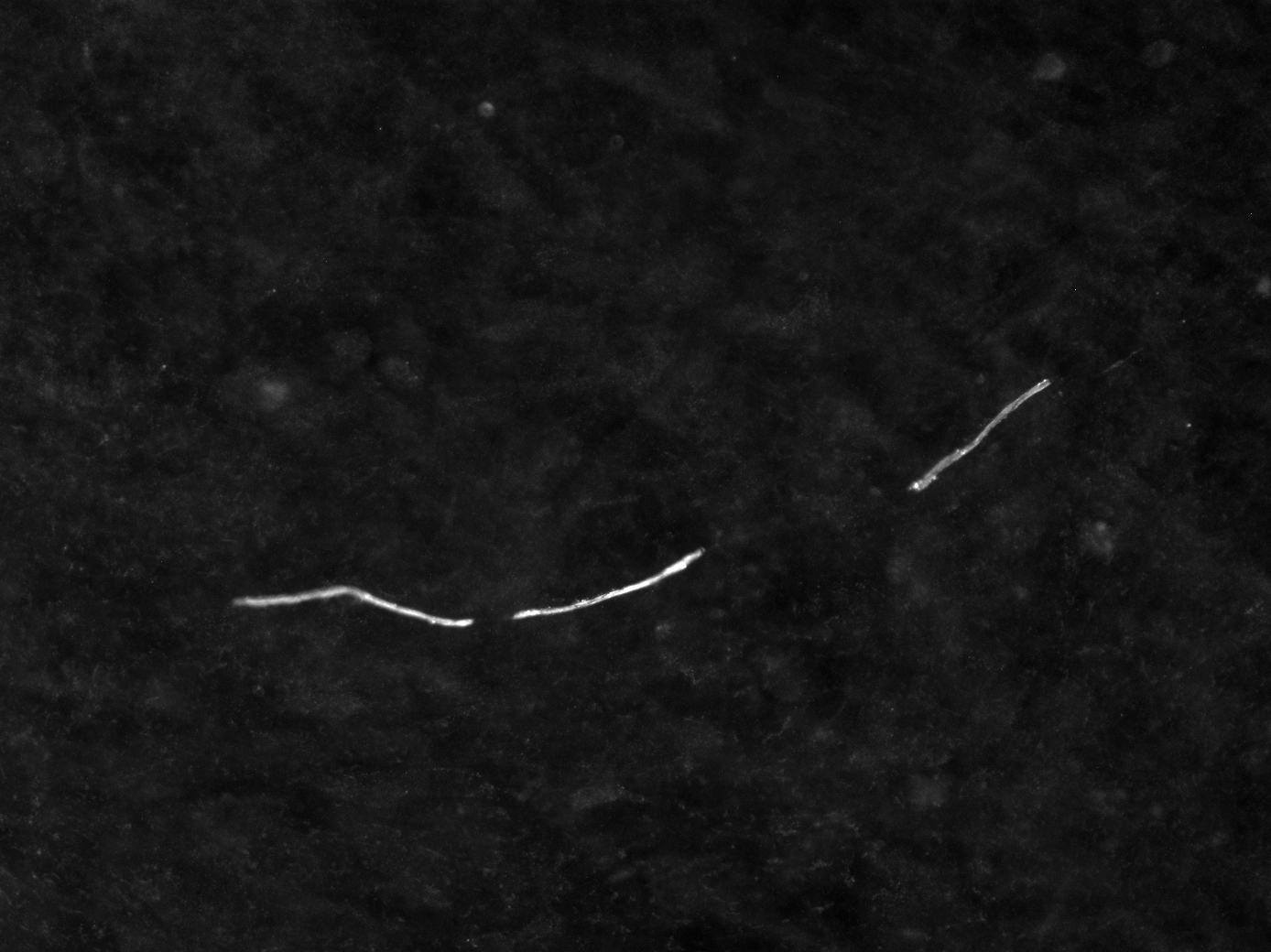

Supplement: Supplementary file 4 — Source Data Fig. 4 [file 44321_2023_19_MOESM4_ESM.zip › Figure4/4B_Images/CMT_CTRL_MBP_.tif]

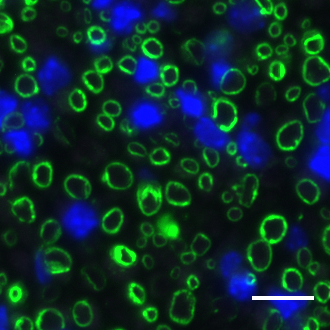

Supplement: Supplementary file 5 — Source Data Fig. 5 [file 44321_2023_19_MOESM5_ESM.zip › Figure5/5E_Images/C61_Composite_10e╠¿m.tif]

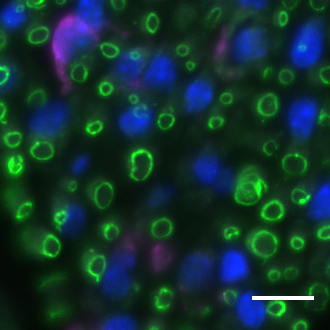

Supplement: Supplementary file 5 — Source Data Fig. 5 [file 44321_2023_19_MOESM5_ESM.zip › Figure5/5E_Images/PTENhxC61_Composite_10e╠¿m.tif]

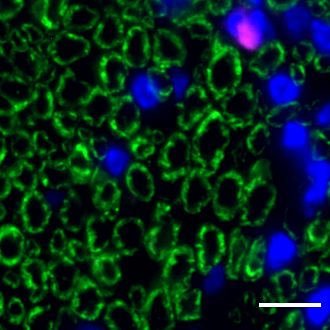

Supplement: Supplementary file 5 — Source Data Fig. 5 [file 44321_2023_19_MOESM5_ESM.zip › Figure5/5E_Images/WT_Composite_10e╠¿m.tif]

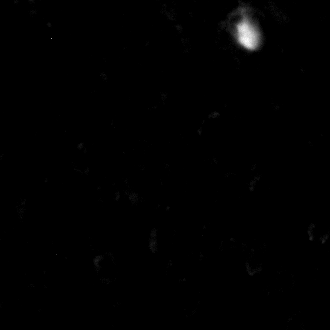

Supplement: Supplementary file 5 — Source Data Fig. 5 [file 44321_2023_19_MOESM5_ESM.zip › Figure5/5E_Images/WT_P-S6.tif]

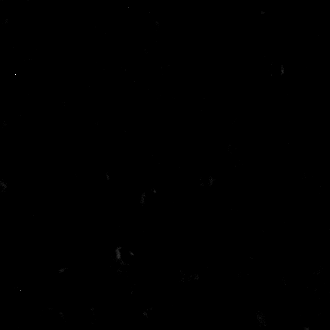

Supplement: Supplementary file 5 — Source Data Fig. 5 [file 44321_2023_19_MOESM5_ESM.zip › Figure5/5E_Images/C61_P-S6.tif]

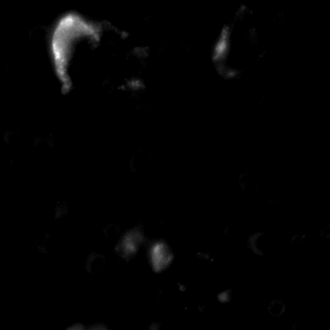

Supplement: Supplementary file 5 — Source Data Fig. 5 [file 44321_2023_19_MOESM5_ESM.zip › Figure5/5E_Images/PTENhxC61_P-S6.tif]

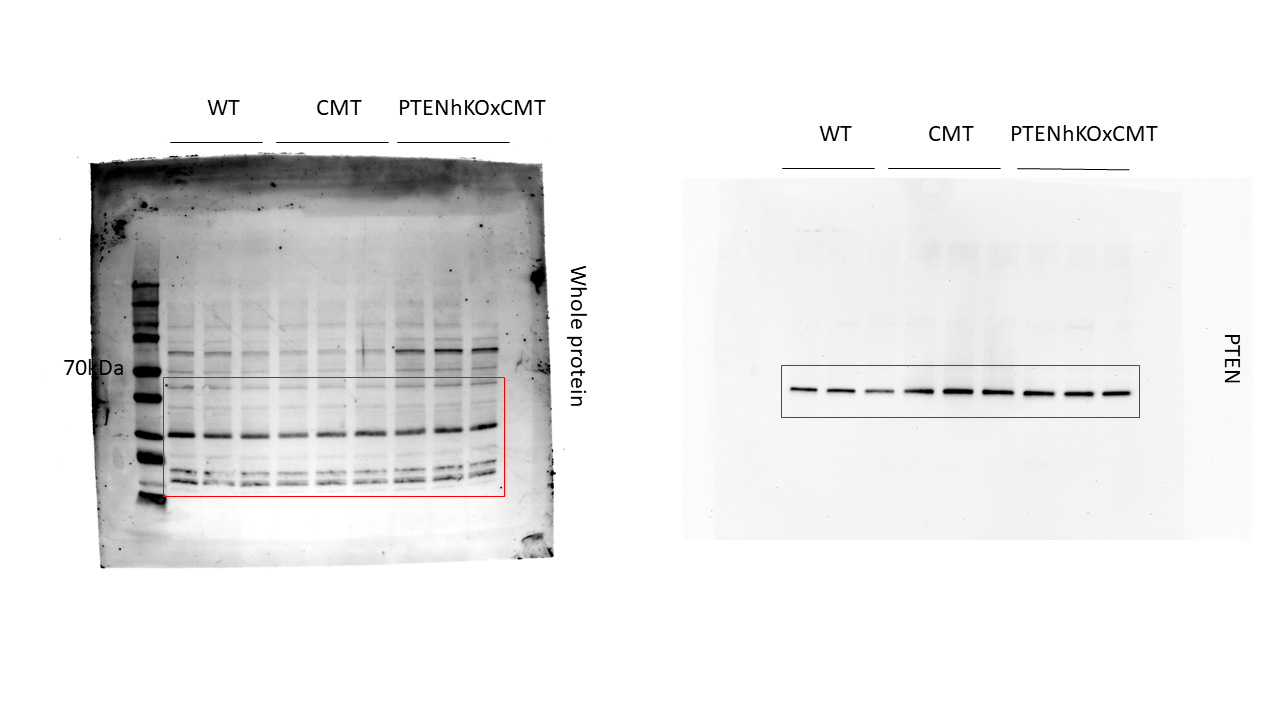

Supplement: Supplementary file 5 — Source Data Fig. 5 [file 44321_2023_19_MOESM5_ESM.zip › Figure5/5C_Blots/Blots_PTEN.TIF]

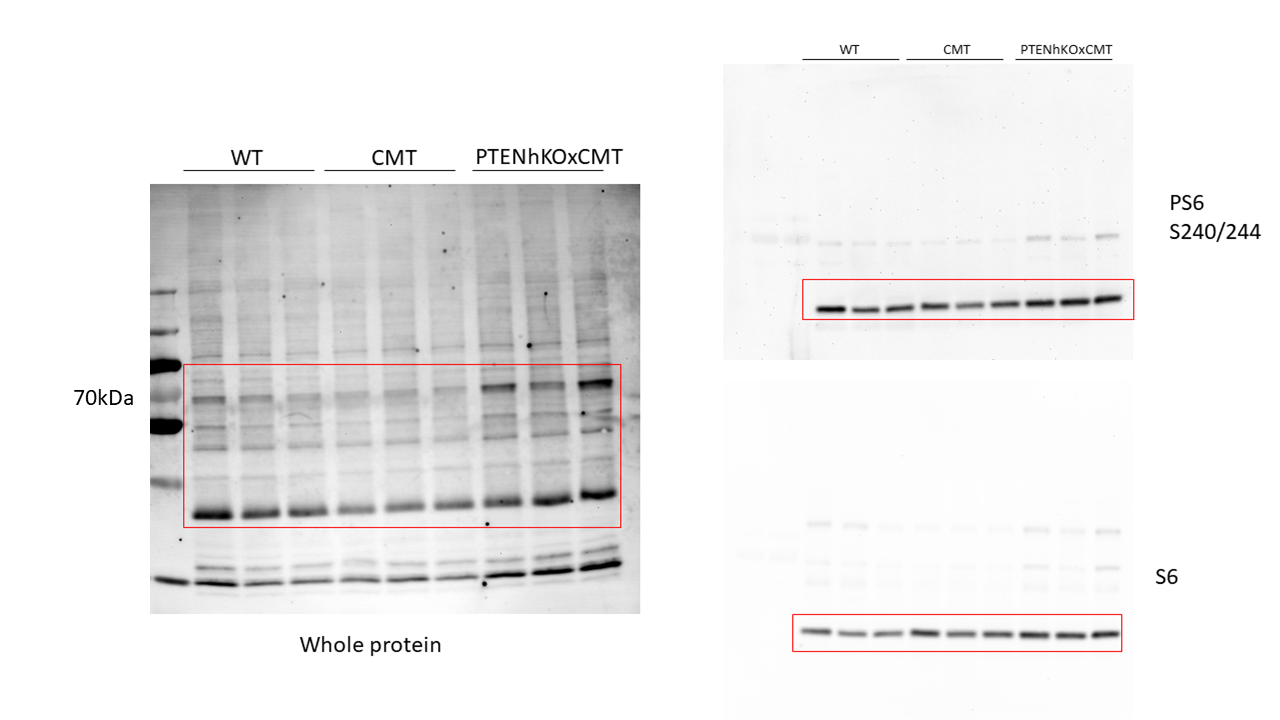

Supplement: Supplementary file 5 — Source Data Fig. 5 [file 44321_2023_19_MOESM5_ESM.zip › Figure5/5C_Blots/Blots_S6.TIF]

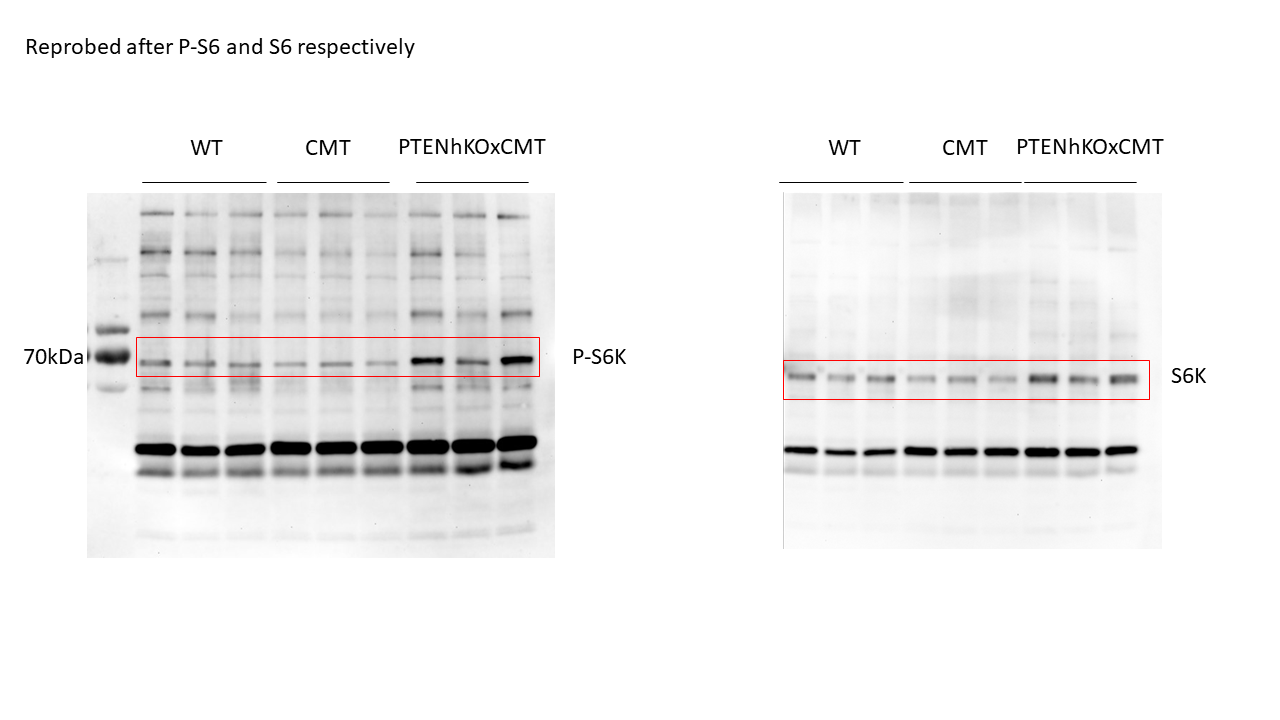

Supplement: Supplementary file 5 — Source Data Fig. 5 [file 44321_2023_19_MOESM5_ESM.zip › Figure5/5C_Blots/Blots_S6K.TIF]

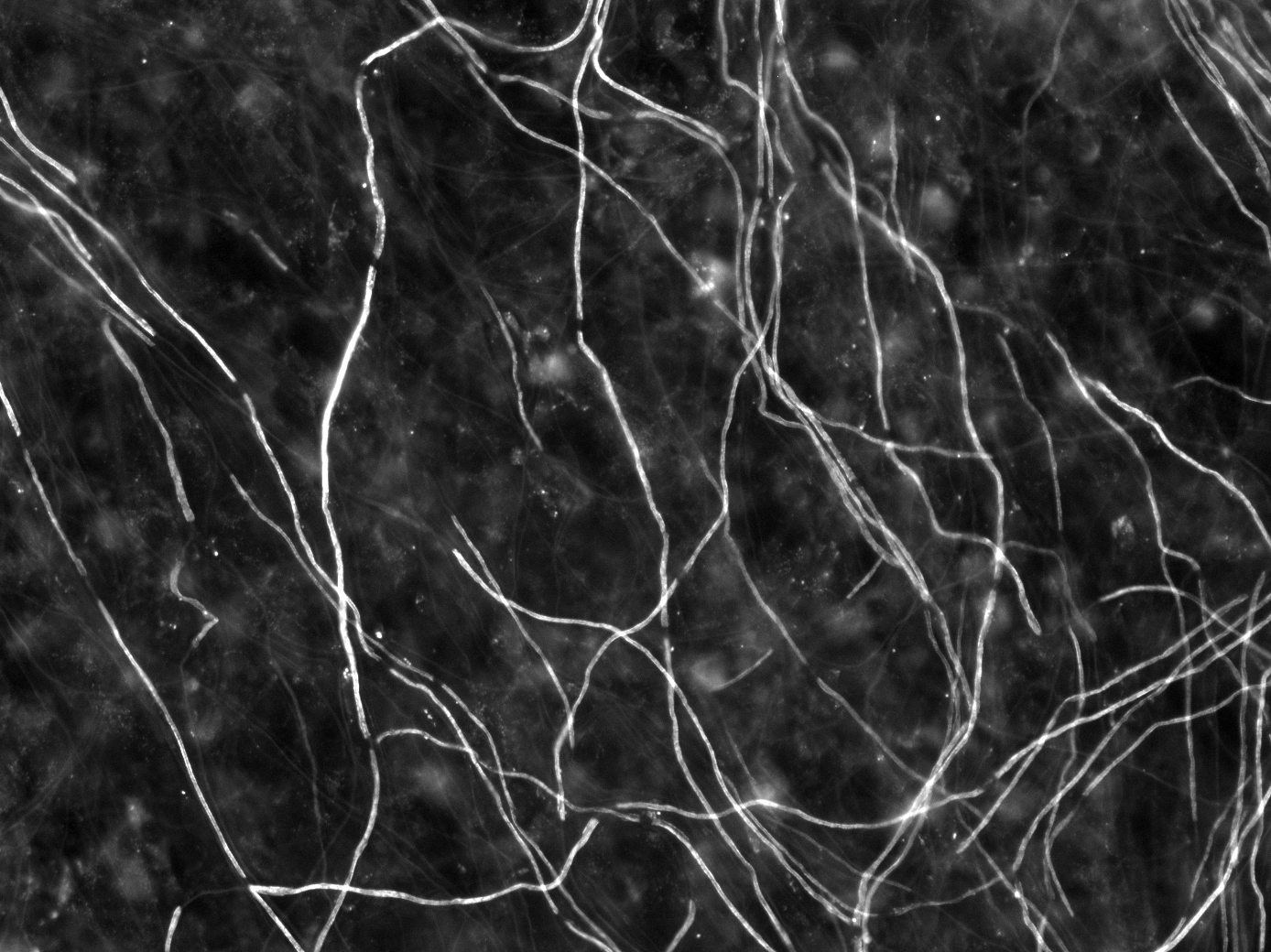

Supplement: Supplementary file 5 — Source Data Fig. 5 [file 44321_2023_19_MOESM5_ESM.zip › Figure5/5F_Images/MBP_WT.tif]

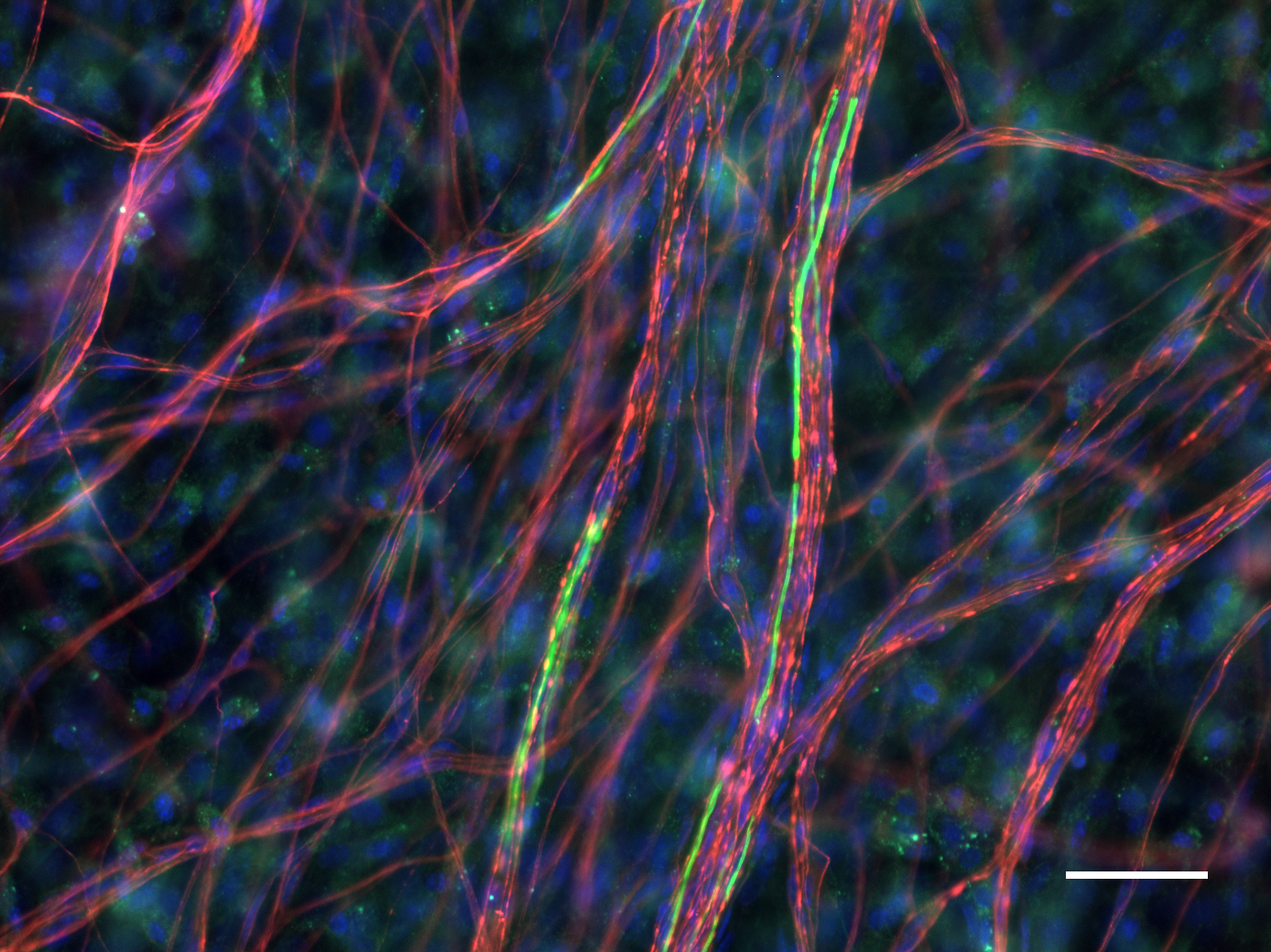

Supplement: Supplementary file 5 — Source Data Fig. 5 [file 44321_2023_19_MOESM5_ESM.zip › Figure5/5F_Images/Composite_C61_50e╠¿m.tif]

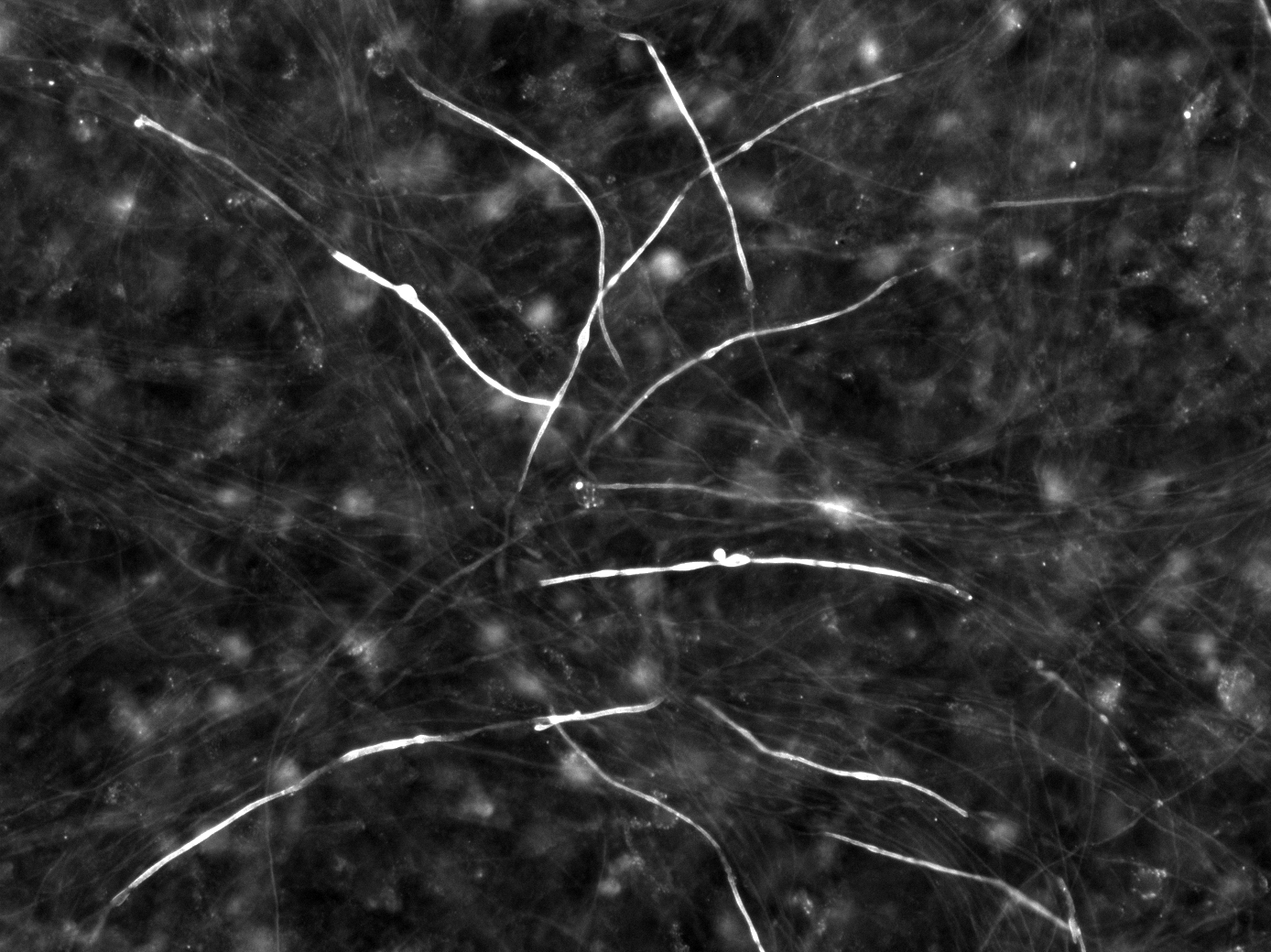

Supplement: Supplementary file 5 — Source Data Fig. 5 [file 44321_2023_19_MOESM5_ESM.zip › Figure5/5F_Images/MBP_hetKOxC61_50e╠¿m.tif]

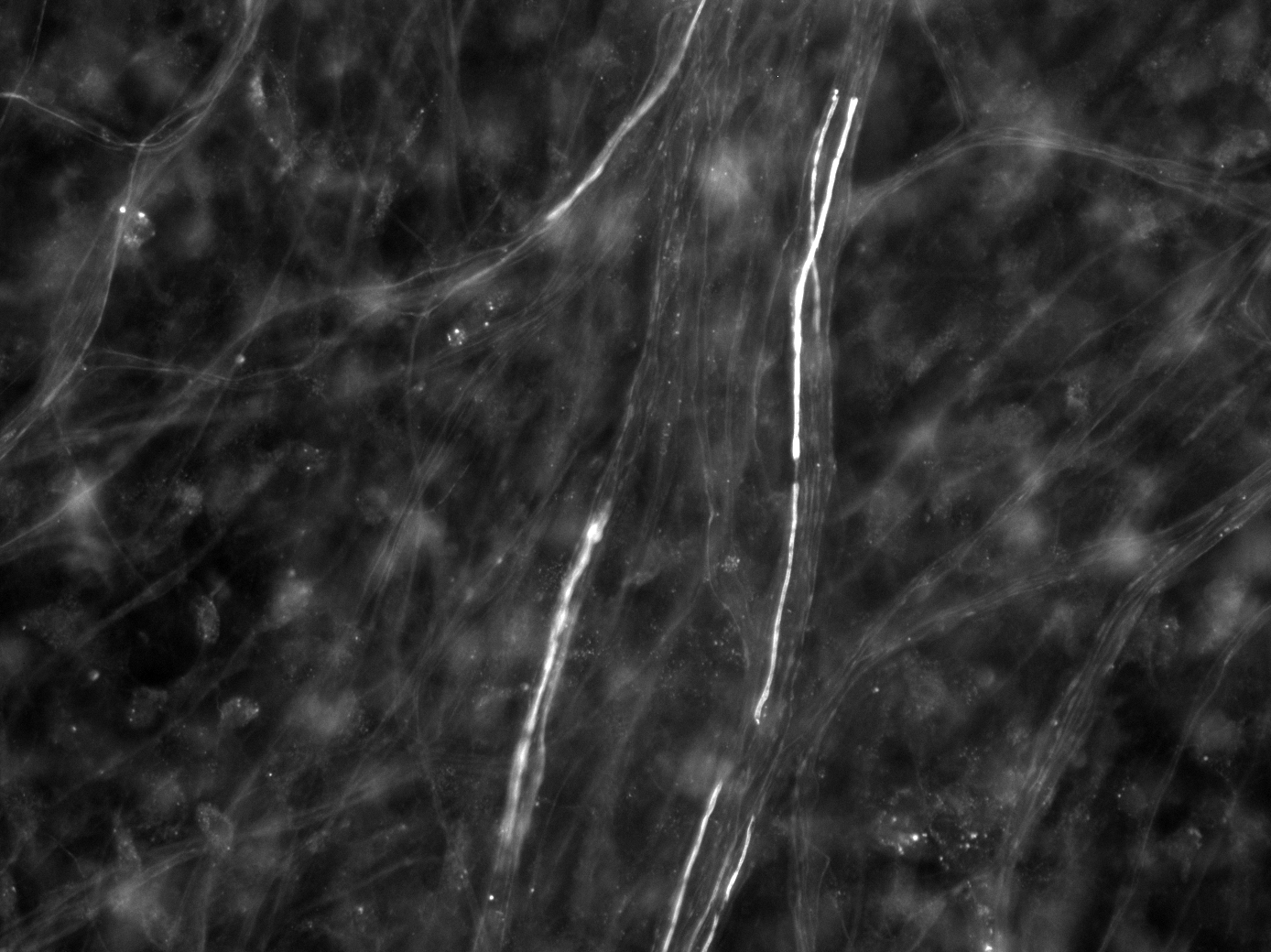

Supplement: Supplementary file 5 — Source Data Fig. 5 [file 44321_2023_19_MOESM5_ESM.zip › Figure5/5F_Images/MBP_C61.tif]

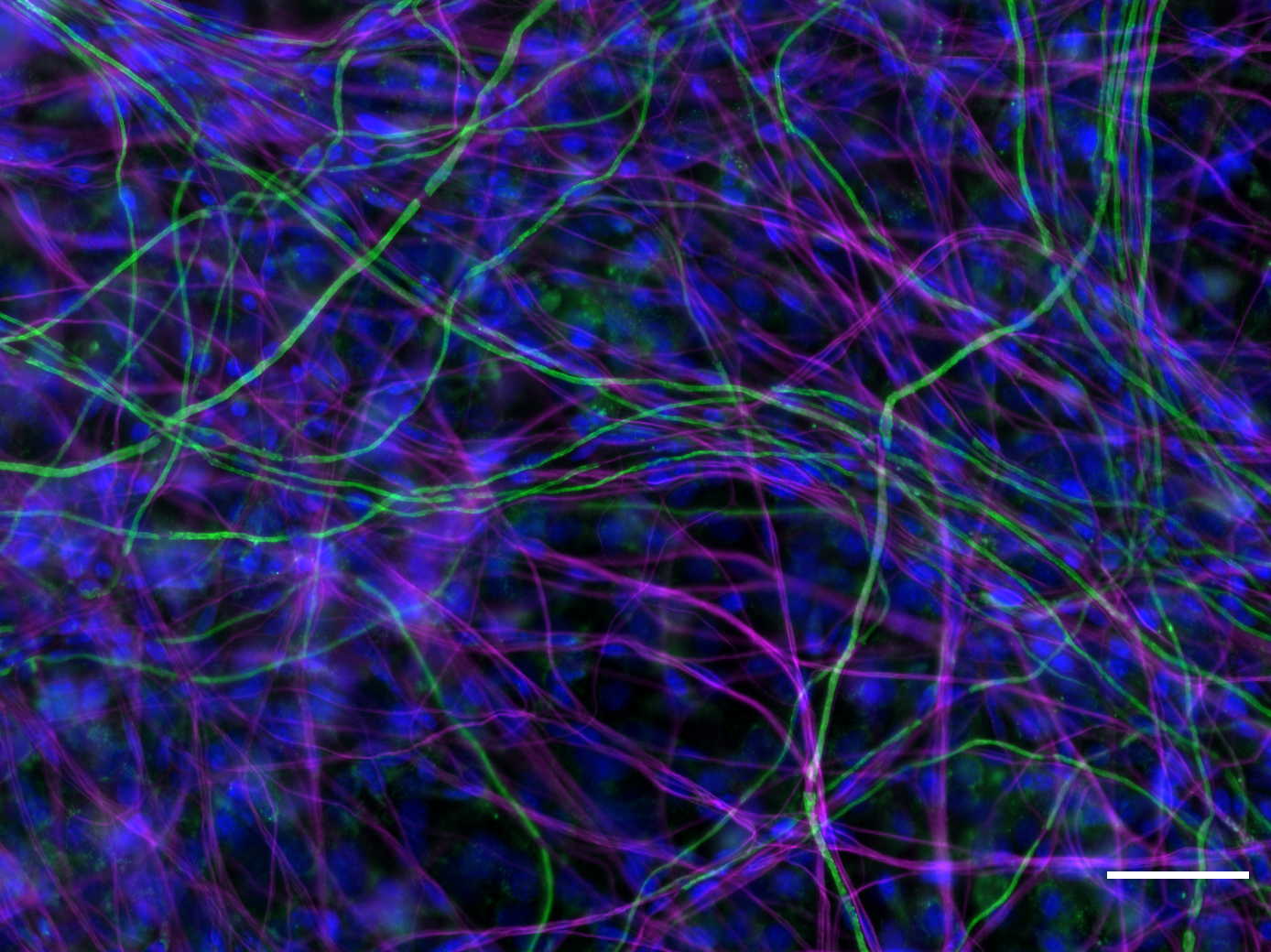

Supplement: Supplementary file 5 — Source Data Fig. 5 [file 44321_2023_19_MOESM5_ESM.zip › Figure5/5F_Images/Composite_hetKO.tif]

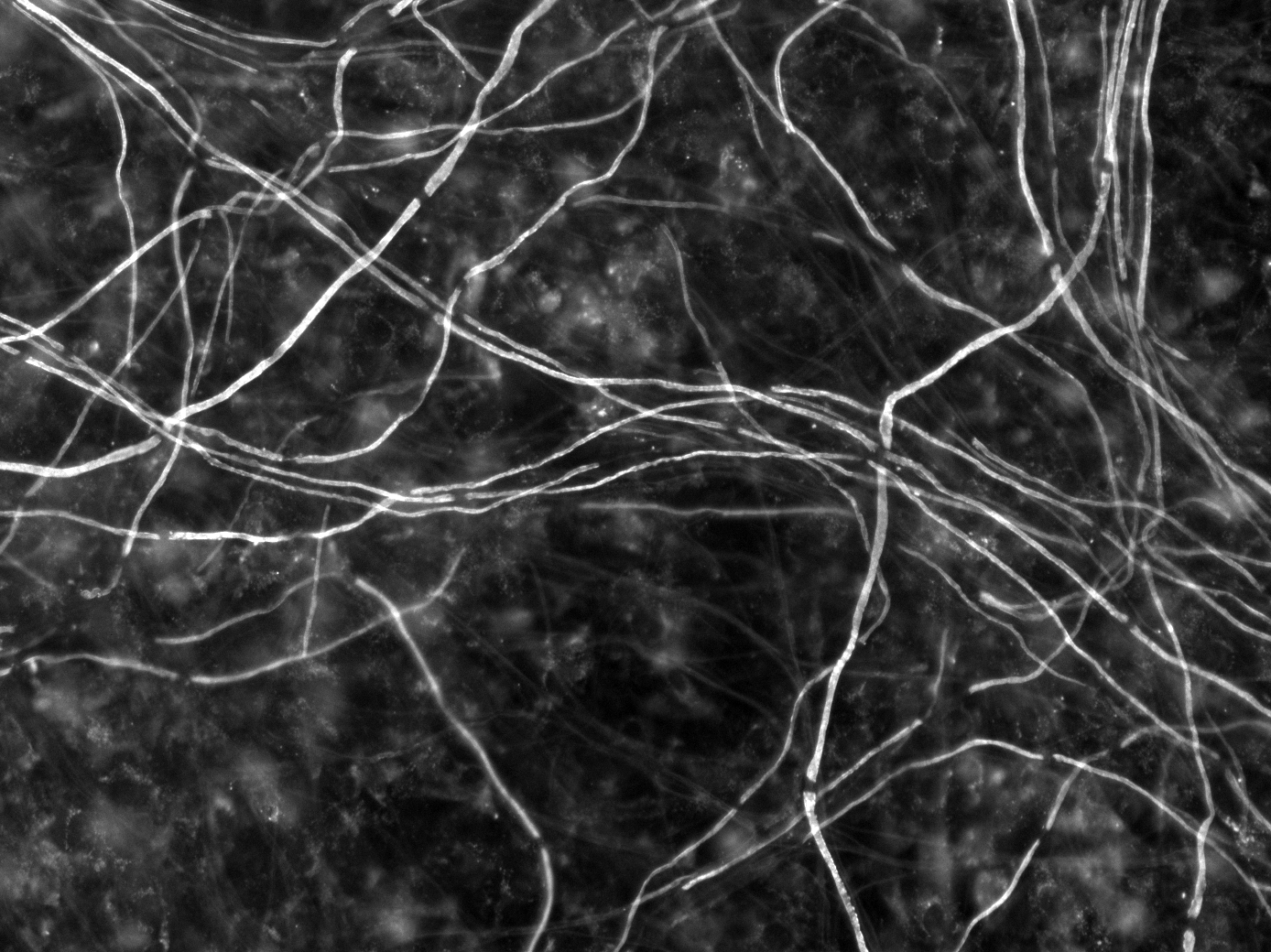

Supplement: Supplementary file 5 — Source Data Fig. 5 [file 44321_2023_19_MOESM5_ESM.zip › Figure5/5F_Images/MBP_hetKO.tif]

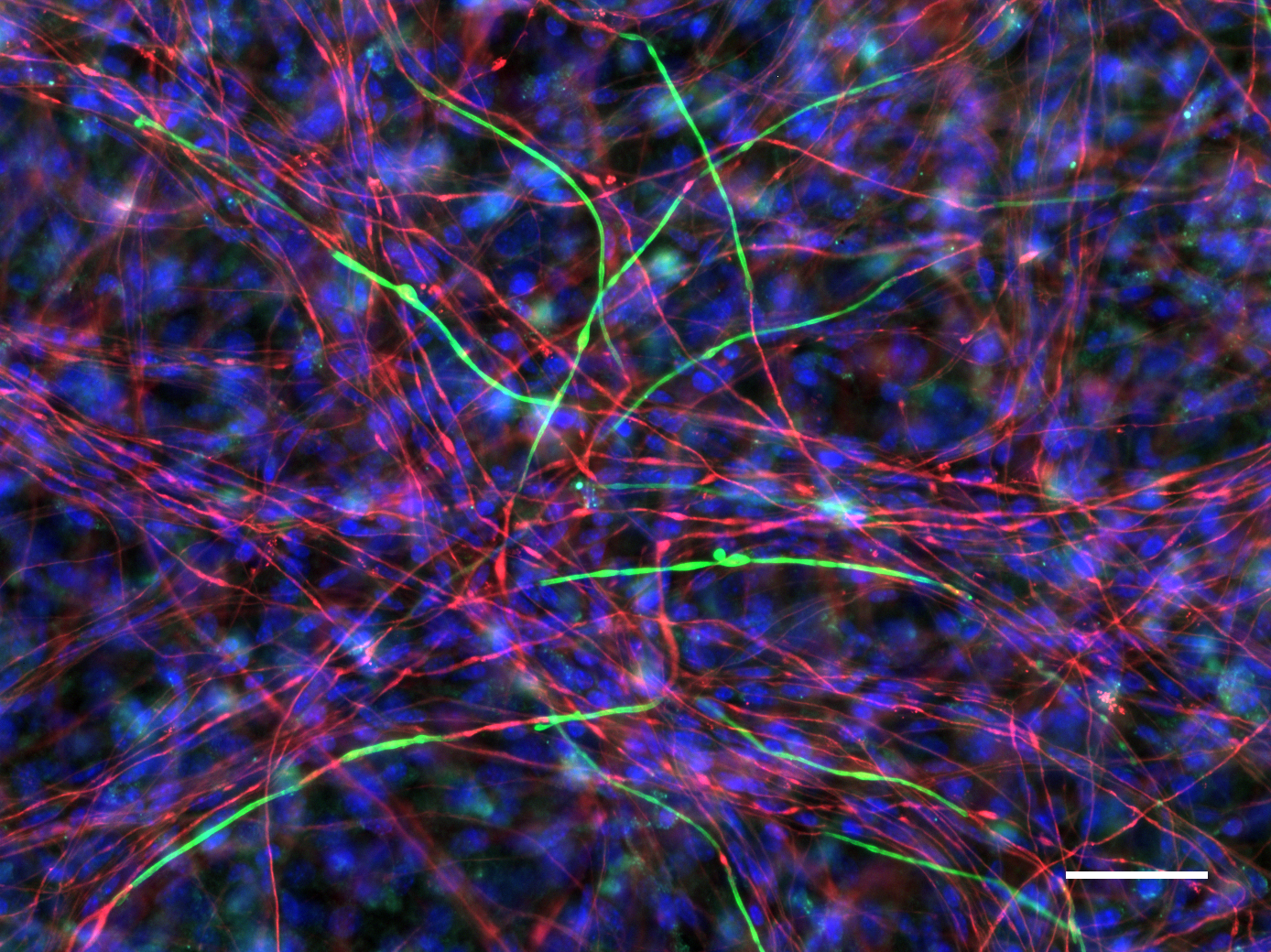

Supplement: Supplementary file 5 — Source Data Fig. 5 [file 44321_2023_19_MOESM5_ESM.zip › Figure5/5F_Images/Composite_hetKOxC61.tif]

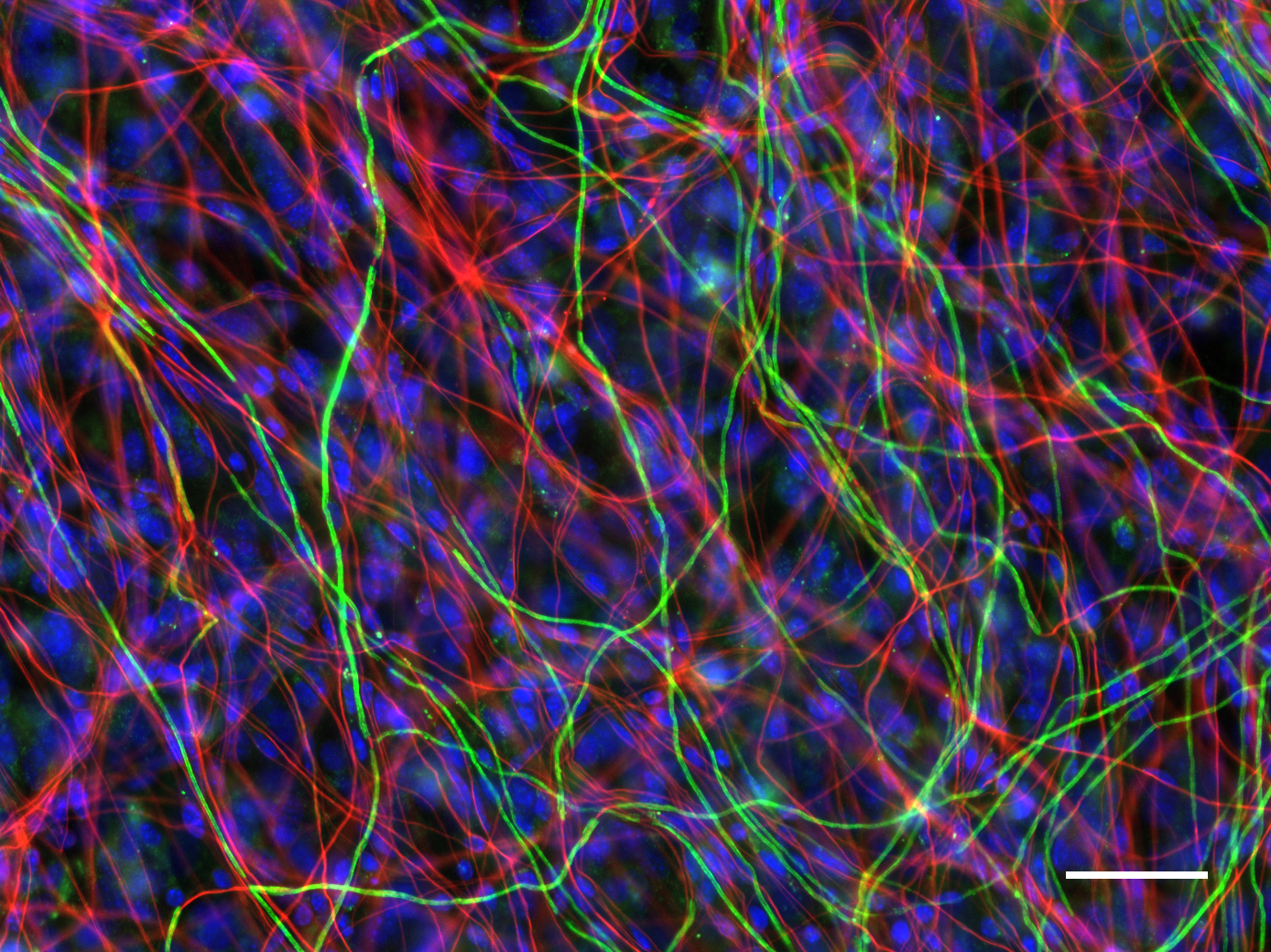

Supplement: Supplementary file 5 — Source Data Fig. 5 [file 44321_2023_19_MOESM5_ESM.zip › Figure5/5F_Images/Composite_WT_50e╠¿m.tif]

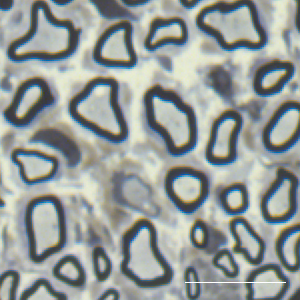

Supplement: Supplementary file 6 — Source Data Fig. 6 [file 44321_2023_19_MOESM6_ESM.zip › Figure6/6A_Images/hKOxC61.tif]

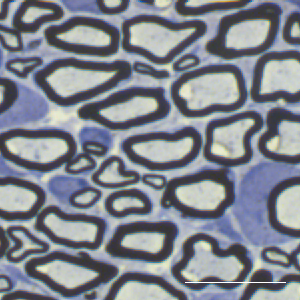

Supplement: Supplementary file 6 — Source Data Fig. 6 [file 44321_2023_19_MOESM6_ESM.zip › Figure6/6A_Images/WT.tif]

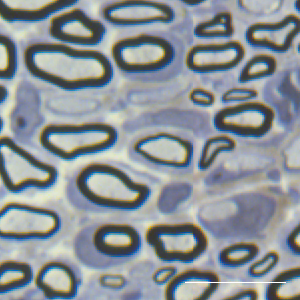

Supplement: Supplementary file 6 — Source Data Fig. 6 [file 44321_2023_19_MOESM6_ESM.zip › Figure6/6A_Images/C61.tif]

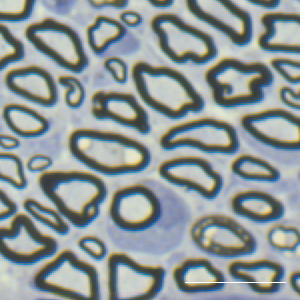

Supplement: Supplementary file 6 — Source Data Fig. 6 [file 44321_2023_19_MOESM6_ESM.zip › Figure6/6A_Images/hetKO.tif]

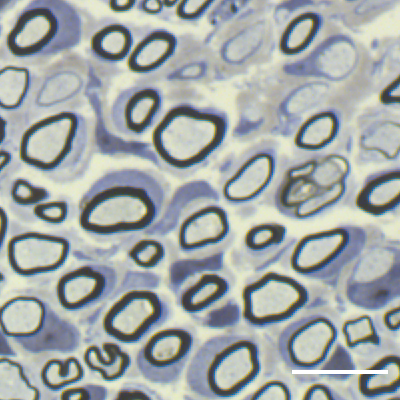

Supplement: Supplementary file 7 — Source Data Fig. 7 [file 44321_2023_19_MOESM7_ESM.zip › Figure7/7C_Image/BspBild_CMT_p18.tif]

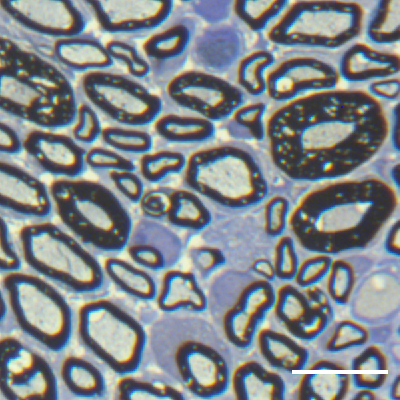

Supplement: Supplementary file 7 — Source Data Fig. 7 [file 44321_2023_19_MOESM7_ESM.zip › Figure7/7C_Image/BspBild_PMPZp18.tif]

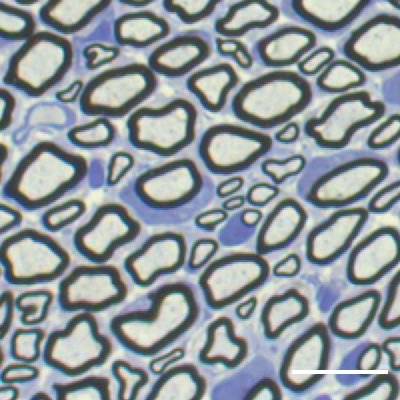

Supplement: Supplementary file 7 — Source Data Fig. 7 [file 44321_2023_19_MOESM7_ESM.zip › Figure7/7C_Image/BspBild_WT_p18.tif]
