# Supplementary material for: Targeting PI3K/Akt/mTOR signaling in rodent models of PMP22 gene-dosage diseases
Source: EMBO Mol Med. 2024 Feb 21;16(3):616–40. doi: 10.1038/s44321-023-00019-5 (PMC10940316; doi:10.1038/s44321-023-00019-5)
Supplement: Supplementary file 9 — Expanded View Figures [file 44321_2023_19_MOESM9_ESM.pdf]

Expanded View Figures

Figure EV1. PMP22 gene dosage dependent alteration of PTEN abundance in *PMP22*$^{+/-}$ HNPP mice and *PMP22*^{tg} CMT1A rat sciatic nerve lysates.

(**A**) Western Blot analysis showing a decrease of PTEN protein levels in sciatic nerve lysates of *Pmp22*$^{+/-}$ mice at 9 weeks ($n = 4$, left panel) and at postnatal day 21 ($n = 4$, right panel) compared to wildtype (WT) control. Fast green whole protein staining was used as loading control for the quantification. (**B**) Western Blot analysis showing a decrease of PTEN protein levels in sciatic nerve lysates of *Pmp22*^{tg} rats at 9 weeks ($n = 4$, left panel) and at postnatal day 18 ($n = 3$, right panel) compared to wildtype (WT) control. Whole protein staining was used as loading control for the quantification. (**C**) Sciatic nerve semi-thin sections of WT ($n = 3$) and *Pmp22*$^{+/-}$ mice ($n = 3$) at postnatal day 6; myelin aberrations are highlighted with yellow arrowheads (left panel). Quantification shows increased percentage of axons with myelin aberrations in sciatic nerves from PMP22$^{+/-}$ mice (left panel). Scale bar is 50 µm. (**D**) Quadriceps motor nerve semi-thin sections of WT ($n = 3$) and *Pmp22*$^{+/-}$ mice ($n = 3$) at postnatal day 18; myelin aberrations are highlighted with yellow arrowheads (left panel). Quantification shows increased percentage of axons with myelin aberrations in sciatic nerves from PMP22$^{+/-}$ mice (left panel). Scale bar is 25 µm. Data information: Means are displayed ± standard deviation. Statistical analysis was performed using Student's *t* test (*$p < 0.05$, **$p < 0.01$).

© The Author(s)

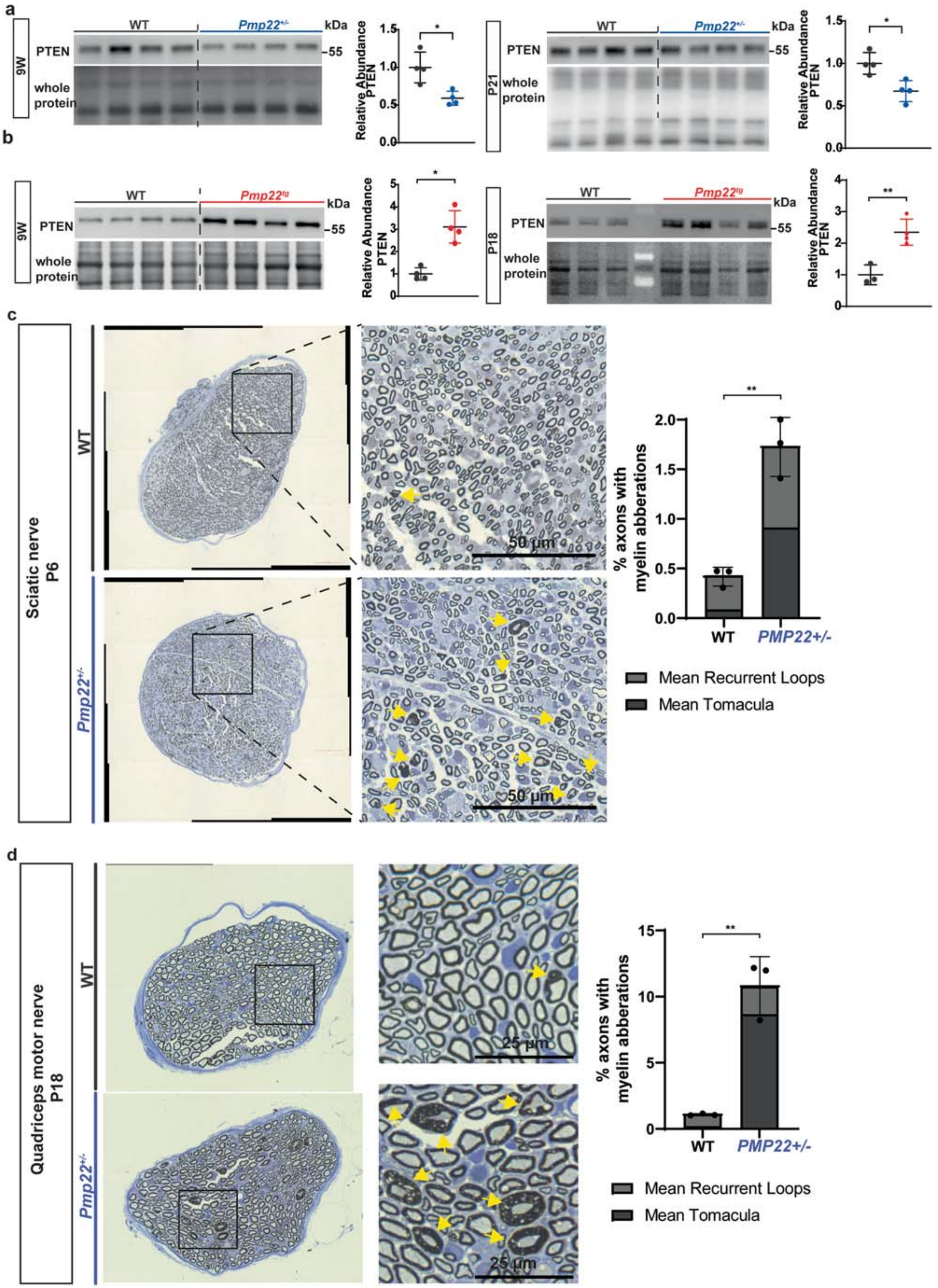
a
9W
WT
Pmp22+/-
kDa
PTEN
-55
whole protein
Relative Abundance PTEN
P21
b
Pmp22tg
P18
c
Sciatic nerve P6
50 µm
% axons with myelin abberations
WT
PMP22+/-
Mean Recurrent Loops
Mean Tomacula
d
Quadriceps motor nerve P18
25 µm

 © The Author(s)

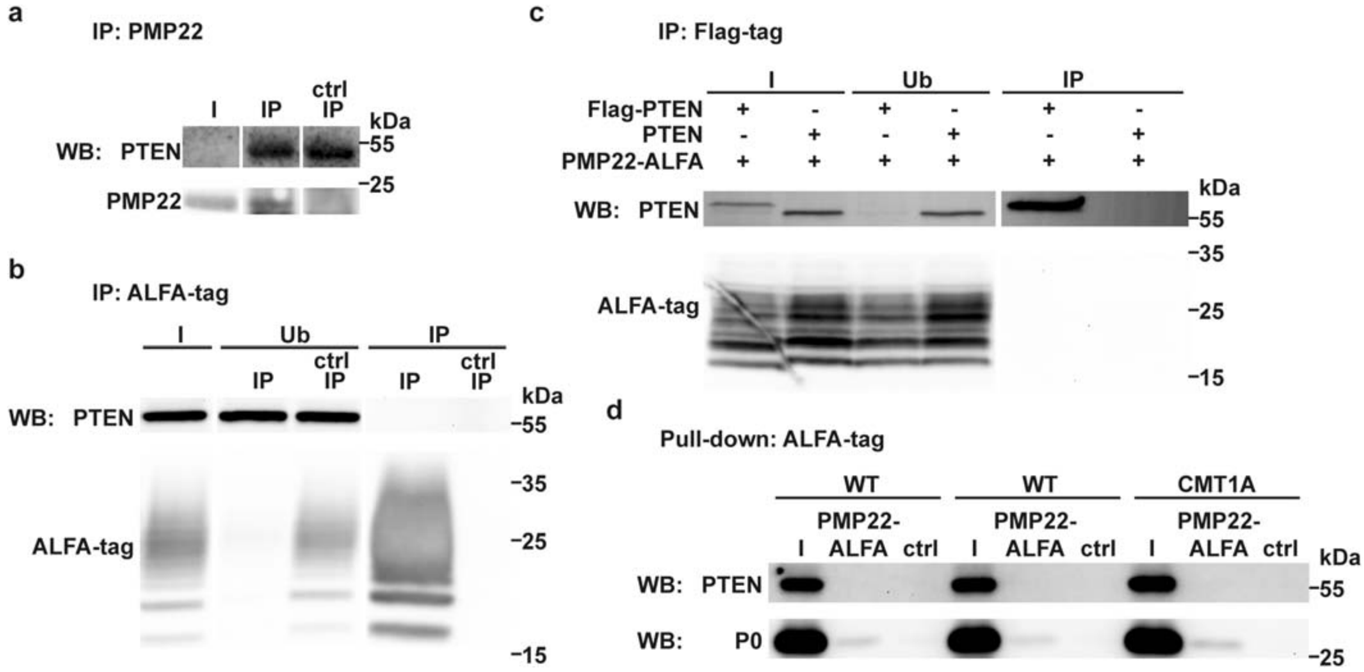

Figure EV2. No evidence for molecular interaction between PMP22 and PTEN in peripheral nerve or in cell culture.

(**A**) Immunoprecipitation of PMP22 from rat sciatic nerve (P18). Western Blot (WB) analysis shows unspecific binding of PTEN, while PMP22 was specifically detected in PMP22 immunoprecipitation eluate (IP) and not in control eluate (ctrl IP). Input nerve homogenate (I) was diluted 50× for WB analysis. (**B**) Immunoprecipitation from HEK293T cells after transfection of PMP22-ALFA. WB analysis shows endogenous PTEN in the cell lysate (I) and in the supernatant after the binding step (unbound (Ub)), but not in the immunoprecipitation eluate. I and Ub were diluted 10× for WB analysis. (**C**) Immunoprecipitation from HEK293T cells after co-transfection of PMP22-ALFA with FLAG-PTEN or untagged PTEN. WB analysis shows specific immunoprecipitation of FLAG-PTEN but not of PMP22-ALFA or untagged PTEN. (**D**) Pull-down assay on rat sciatic nerve (P18) using purified PMP22-ALFA as prey. WB analysis shows PTEN in the nerve lysate (I) but not in the PMP22-ALFA eluate (PMP22-ALFA) or control eluate (ctrl) in both WT and CMT1A, while P0 was pulled down by PMP22-ALFA. I was diluted 3.33× for WB analysis.

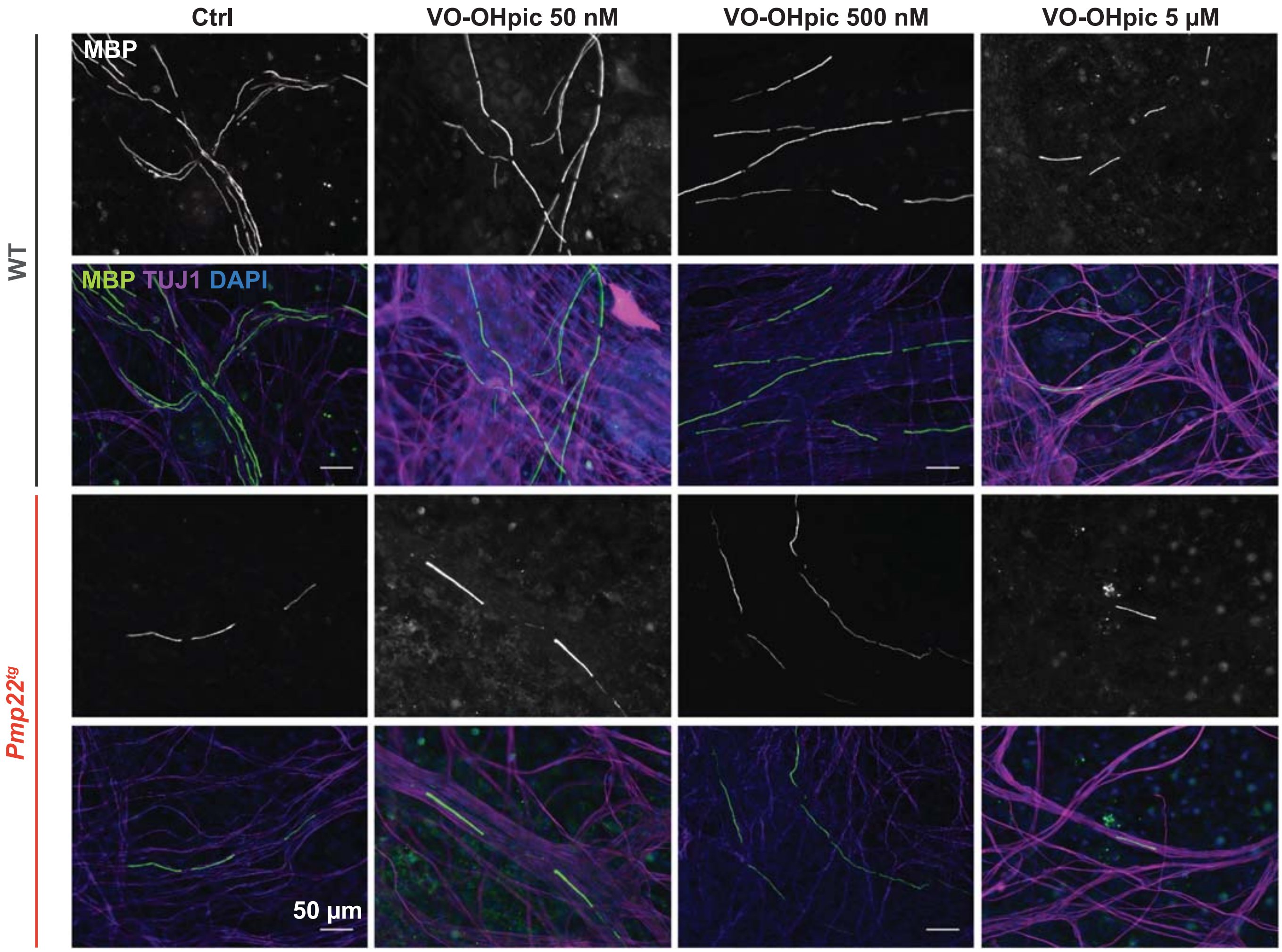

Figure EV3. Dose-dependent response of myelination upon PTEN inhibition in *Pmp22*^{tg} co-cultures in vitro.

Example images of SC-DRG co-cultures from wildtype (WT) and *Pmp22*^{tg} rats, treated with different dosages of the PTEN inhibitor VO-OHpic for 14 days. The number of myelinated segments (MBP; gray/green) decreases in WT cultures with increasing inhibitor dosage. In *Pmp22*^{tg} co-cultures an increase is observed up to 500 nM VO-OHpic but a decrease with 5 μM VO-OHpic. Scale bar is 50 μm. Images for 500 nM VO-OHpic treatment are the same as used in Fig. 4B.

 © The Author(s)

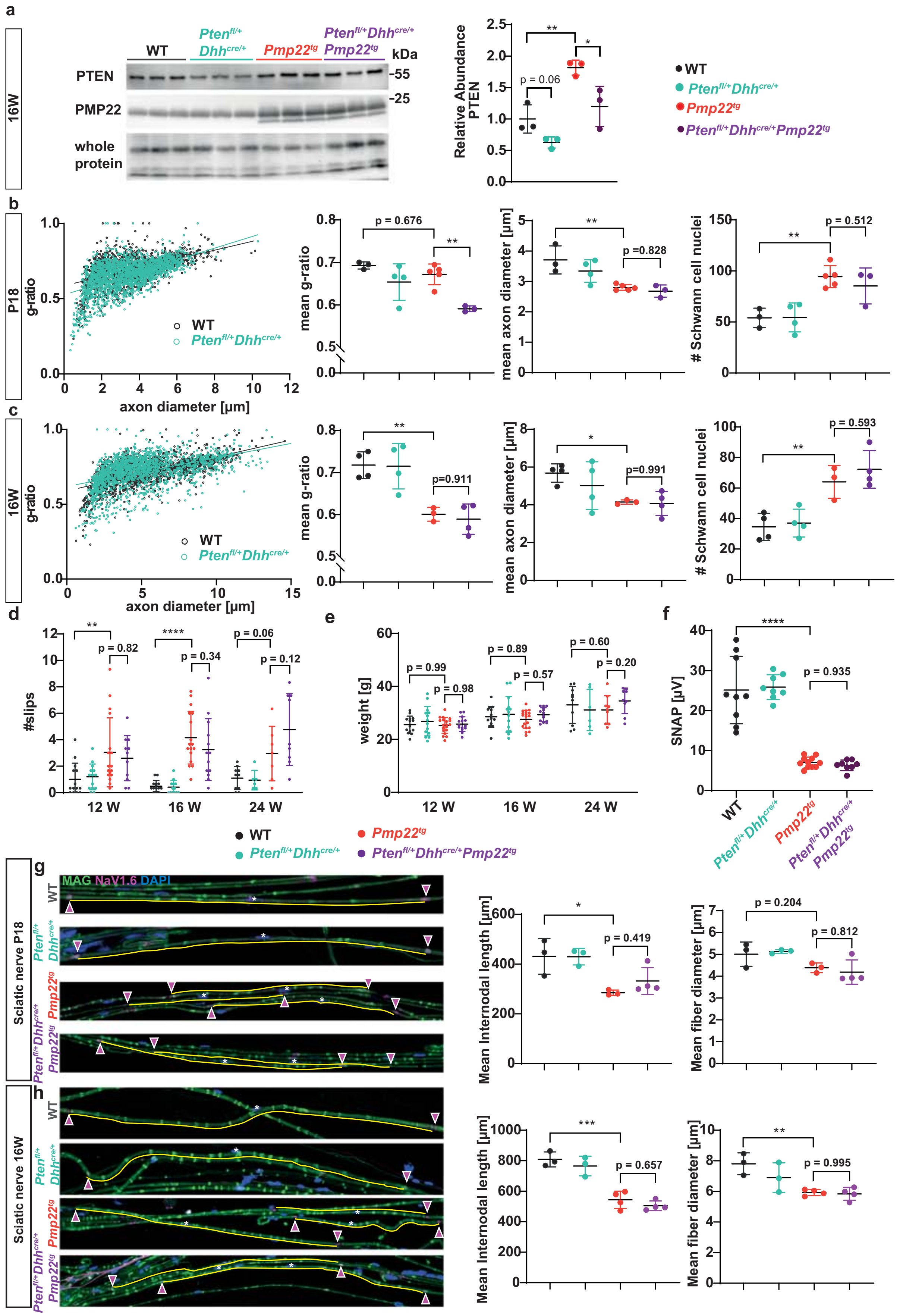

Figure EV4. Unaltered internodal length and myelin sheath thickness in $Pten^{fl/+}Dhh^{cre/+}$ mice.

(**A**) Western Blot analysis shows PTEN and PMP22 protein amounts in whole sciatic nerve lysates from 16 weeks old WT, PTEN heterozygous knockout ($Pten^{fl/+}Dhh^{cre/+}$), CMT1A ($Pmp22^{tg}$) and double mutant ($Pten^{fl/+}Dhh^{cre/+}Pmp22^{tg}$) mice using whole protein staining as loading control. (**B**) *G*-ratio plotted against axon diameter of wildtype (WT, gray) and $Pten^{fl/+}Dhh^{cre/+}$ (turquoise) femoral nerves at P18. Mean *g*-ratio is unaltered in $Pten^{fl/+}Dhh^{cre/+}$ and $Pmp22^{tg}$ mice compared to WT controls and decreased in $Pten^{fl/+}Dhh^{cre/+}Pmp22^{tg}$ mice (left panel). Mean axon diameters are reduced in $Pmp22^{tg}$ and $Pten^{fl/+}Dhh^{cre/+}Pmp22^{tg}$ mice (middle panel). The number of Schwann cell nuclei per femoral nerve cross section is increased in $Pmp22^{tg}$ and $Pten^{fl/+}Dhh^{cre/+}Pmp22^{tg}$ mice. WT $n = 3$, $Pten^{fl/+}Dhh^{cre/+}$ $n = 4$, $Pmp22^{tg}$ $n = 5$ and $Pten^{fl/+}Dhh^{cre/+}Pmp22^{tg}$ $n = 3$ animals. (**C**) *G*-ratio plotted against axon diameter of WT (gray) and $Pten^{fl/+}Dhh^{cre/+}$ (turquoise) femoral nerves at 16 weeks of age. Mean g-ratio is unaltered in $Pten^{fl/+}Dhh^{cre/+}$ mice compared to WT controls and decreased in $Pmp22^{tg}$ and $Pten^{fl/+}Dhh^{cre/+}Pmp22^{tg}$ mice (left panel). Mean axon diameters are reduced in $Pmp22^{tg}$ and $Pten^{fl/+}Dhh^{cre/+}Pmp22^{tg}$ mice (middle panel). The number of Schwann cell nuclei per femoral nerve cross section is increased in $Pmp22^{tg}$ and $Pten^{fl/+}Dhh^{cre/+}Pmp22^{tg}$ mice. WT $n = 4$, $Pten^{fl/+}Dhh^{cre/+}$ $n = 4$, $Pmp22^{tg}$ $n = 3$ and $Pten^{fl/+}Dhh^{cre/+}Pmp22^{tg}$ $n = 4$ animals. (**D**) The number of slips on the elevated beam is similarly increased in $Pmp22^{tg}$ and $Pten^{fl/+}Dhh^{cre/+}Pmp22^{tg}$ mice compared to wildtype controls at all time points. Behavioral analysis was done at 12, 16 and 24 weeks of age. WT $n = 10$–14, $Pten^{fl/+}Dhh^{cre/+}$ $n = 6$–14, $Pmp22^{tg}$ $n = 9$–19 and $Pten^{fl/+}Dhh^{cre/+}Pmp22^{tg}$ $n = 9$–14 mice were analyzed. (**E**) Neither the weight of $Pmp22^{tg}$ nor $Pten^{fl/+}Dhh^{cre/+}Pmp22^{tg}$ mice is altered compared to wildtype controls at 12, 16 and 24 weeks of age. WT $n = 10$–14, $Pten^{fl/+}Dhh^{cre/+}$ $n = 6$–14, $Pmp22^{tg}$ $n = 9$–19 and $Pten^{fl/+}Dhh^{cre/+}Pmp22^{tg}$ n = 9-14 mice were analyzed. (**F**) Sensory nerve action potential amplitudes (SNAP) are decreased in the tail of $Pmp22^{tg}$ and $Pten^{fl/+}Dhh^{cre/+}Pmp22^{tg}$ mice compared to wildtype controls. For electrophysiology measurements WT $n = 10$, $Pten^{fl/+}Dhh^{cre/+}$ $n = 8$, $Pmp22^{tg}$ $n = 11$ and $Pten^{fl/+}Dhh^{cre/+}Pmp22^{tg}$ $n = 8$ mice were analyzed. (**G**) Example images of teased fiber preparations of WT, $Pten^{fl/+}Dhh^{cre/+}$, $Pmp22^{tg}$ and $Pten^{fl/+}Dhh^{cre/+}Pmp22^{tg}$ double mutants stained for MAG (green), NaV1.6 (magenta) and DAPI (blue) at P18. Internodes between two nodes (magenta arrowheads) are underlined in yellow and respective Schwann cell nuclei are marked by white stars. Mean internodal length (left panel) is significantly reduced in $Pmp22^{tg}$ teased fibers compared to wildtype controls at P18, whereas $Pten^{fl/+}Dhh^{cre/+}Pmp22^{tg}$ mice do not differ in internodal length compared to $Pmp22^{tg}$ mice. Mean fiber diameters are not significantly altered (right panel). Analysis was performed on 100 internodes of $n = 3$–4 animals per group. (**H**) Example images of teased fiber preparations of WT, $Pten^{fl/+}Dhh^{cre/+}$, $Pmp22^{tg}$ and $Pten^{fl/+}Dhh^{cre/+}Pmp22^{tg}$ double mutants stained for MAG (green), NaV1.6 (magenta) and DAPI (blue) at 16 weeks of age. Internodes between two nodes (magenta arrowheads) are underlined in yellow and respective Schwann cell nuclei are marked by white stars. Mean internodal length (left panel) and fiber diameter (right panel) are significantly reduced in $Pmp22^{tg}$ teased fibers compared to wildtype controls at 16 weeks of age, whereas $Pten^{fl/+}Dhh^{cre/+}Pmp22^{tg}$ mice do not differ in internodal length compared to $Pmp22^{tg}$ mice. Analysis was performed on 100 internodes of $n = 3$–4 animals per group. Data information: Means are displayed ± standard deviation. Statistical analysis was done using one-way ANOVA with Sidak's multiple comparison test ($*p \le 0.05$, $**p \le 0.01$, $***p \le 0.001$, $****p \le 0.0001$).

© The Author(s)

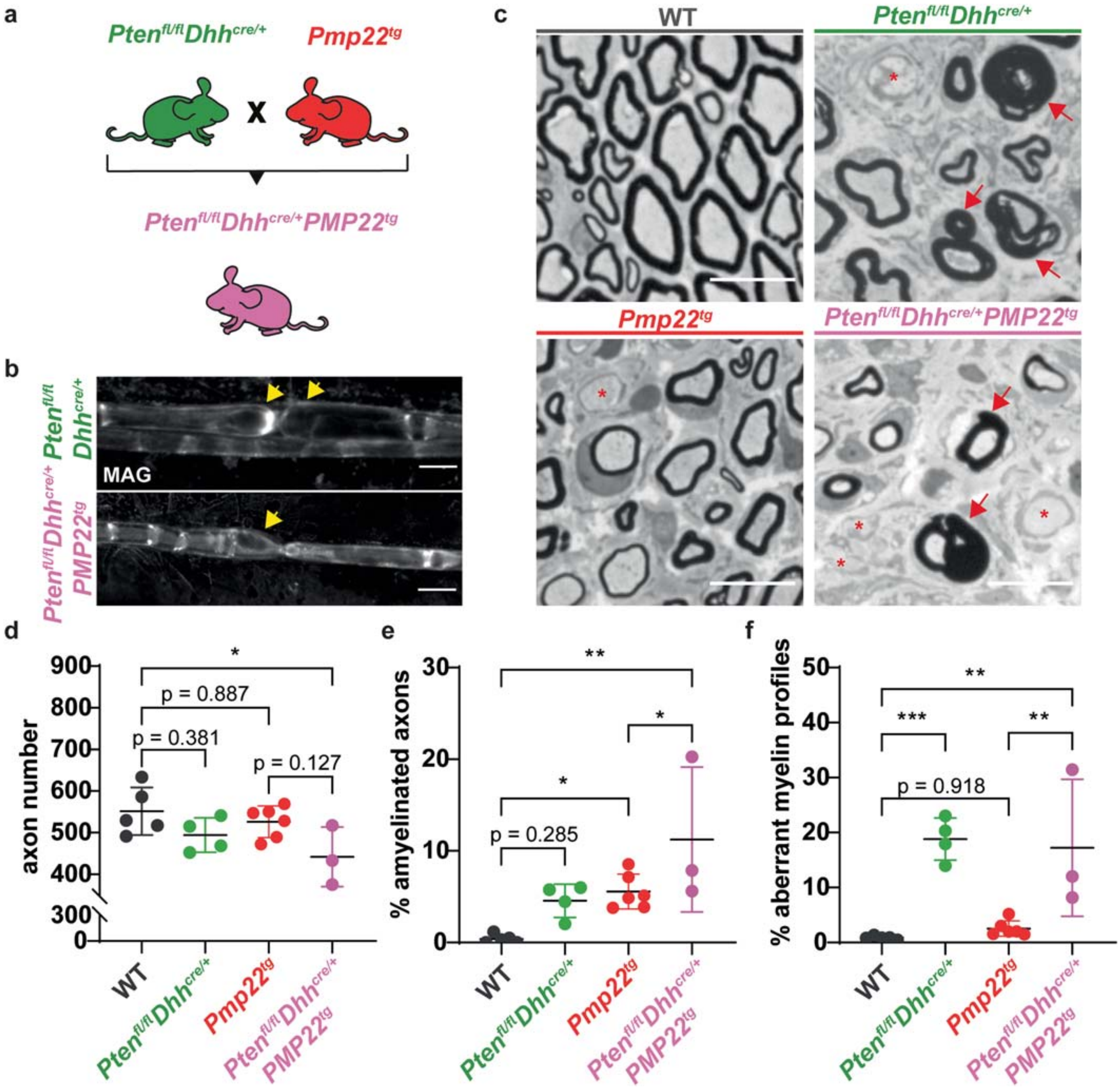

Figure EV5. ***Pten* ablation in *Pmp22^{tg}* mice leads to myelin abnormalities.**

(**A**) Crossing scheme of Schwann cell specific full Pten knockout mice (*Pten$^{fl/fl}$Dhh$^{cre/+}$*) with CMT1A mice (*Pmp22^{tg}*) to generate a full *Pten* knockout in CMT1A mice (*Pten$^{fl/fl}$Dhh$^{cre/+}$Pmp22^{tg}*). (**B**) Teased fiber preparations of 8 weeks old *PTEN$^{fl/fl}$Dhh$^{cre/+}$* (upper panel) and *Pten$^{fl/fl}$Dhh$^{cre/+}$Pmp22^{tg}* mice (lower panel) show focal myelin thickening at paranodal loops as indicated by yellow arrows. Scale bar = 20 µm. (**C**) Semi-thin cross section of femoral nerves from WT, *Pten$^{fl/fl}$Dhh$^{cre/+}$*, *Pmp22^{tg}* and *Pten$^{fl/fl}$Dhh$^{cre/+}$Pmp22^{tg}* mice at 8 weeks of age. Red arrows indicate myelin abnormalities such as outfoldings and tomacula, asterisks indicate amyelinated axons. Scale bar = 10 µm. (**D–F**) Quantification of (**C**) displays reduced axon numbers in *Pten$^{fl/fl}$Dhh$^{cre/+}$Pmp22^{tg}* mice compared to wildtype controls (**D**). The percentage of amyelinated axons is increased in *Pmp22^{tg}* and further elevated in *Pten$^{fl/fl}$Dhh$^{cre/+}$Pmp22^{tg}* mice (**E**). *Pten* depletion alone and in *Pmp22^{tg}* leads to an increase in axons with aberrant myelin profiles (**F**). WT $n = 5$, *PTEN$^{fl/fl}$Dhh$^{cre/+}$* $n = 4$, *Pmp22^{tg}* $n = 6$ and *Pten$^{fl/fl}$Dhh$^{cre/+}$Pmp22^{tg}* $n = 3$ animals were analyzed. Data information: Means are displayed ± standard deviation. Statistical analysis was done using one-way ANOVA with Sidak's multiple comparison test (*$p \leq 0.05$, **$p \leq 0.01$, ***$p \leq 0.001$).
